# Maternal experience alters brain-wide representation of infant cries

**DOI:** 10.64898/2026.06.26.734581

**Published:** 2026-06-28

**Authors:** Briana R. McRae, Dianne-Lee K.D. Ferguson, Kalei Swanier, Genevera Allen, Bianca J. Marlin

**Affiliations:** 1 Mortimer B. Zuckerman Mind Brain and Behavior Institute, Columbia University; New York, 10027, USA.; 2 Howard Hughes Medical Institute, Columbia University, New York, NY, USA.; 3 Department of Neuroscience, Columbia University; New York, 10032, USA.; 4 Department of Psychology, Columbia University; New York, 10032, USA.; 5 Department of Statistics, Columbia University; New York, 10032, USA.

## Abstract

Pup calls signal the presence of a mouse infant in need of care (Bernd Haack et al., 1983; Ehret, 2005). Previous work demonstrated that experience-dependent plasticity within the left primary auditory cortex is oxytocin-facilitated and enhances neural and behavioral responses to these distress vocalizations (Marlin et al., 2015). However, whether maternal experience reshapes pup call processing beyond auditory regions remains unknown. Here, we show that both innate and learned maternal experience shape pup call-evoked behavior and brain-wide neural responses. Combining behavioral assays, whole-brain activity mapping, and oxytocinergic projection analyses, we find that pup calls recruit distinct large-scale neural networks in naïve virgins, experienced virgins, and mothers. With more maternal experience we observe a distributed pup call response network that is sparser yet more strongly coactivated, consistent with a more efficient and specialized neural representation of infant cues. We identified an oxytocin-dense circuit that shows an experience-dependent pup call response pattern. Therefore, we propose that experience-dependent refinement of the brain-wide response to pup calls is facilitated by oxytocin. Taken together, our findings reveal that maternal experience refines brain-wide sensory processing to support adaptive caregiving behavior.

## One Sentence

Maternal experience fundamentally reorganizes infant cue processing across the entire brain into a more efficient and specialized caregiving state.

## Introduction

Motherhood requires rapid adaptation to infant-derived sensory cues that guide caregiving behavior. Across mammals, infants communicate their needs through vocalizations, including crying in humans and ultrasonic vocalizations in house mice (*Mus musculus*). These vocal signals are among the most salient sensory cues encountered by caregivers and can powerfully shape maternal behavior.

Mouse pups emit ultrasonic isolation calls when separated from the nest (Ehret, 2005; Liu et al., 2003; Noirot, 1972). These vocalizations drive maternal behavior, guiding caregivers toward displaced offspring and eliciting pup retrieval (Ehret, 2005; Hernandez-Miranda et al., 2017a; Schiavo et al., 2020). Because auditory cues can signal the location of a pup from a distance, pup calls represent highly salient sensory signals for caregivers, even in the absence of pup cues of other sensory modalities (Bernd Haack et al., 1983; McRae et al., 2023). Consistent with this, playback of pup calls alone is sufficient to attract mothers toward a sound source, eliciting the phonotaxis response that marks the initiation of pup retrieval (Ehret, 1987; Schiavo et al., 2020; Sewell, 1970; Tasaka et al., 2020).

Mothers are experts at pup retrieval, even generalizing to retrieve pups that are not their own (Bendesky et al., 2017; Chantrey & Jenkins, 1982; Khadraoui et al., 2022). On the contrary, naïve virgin females typically do not retrieve pups reliably (Carcea et al., 2021; Ehret et al., 1987; Ehret & Koch, 1989; Koch & Ehret, 1989; Marlin et al., 2015; Schiavo et al., 2020). However, following cohousing with a mother and litter for multiple days, virgin females can learn to perform pup retrieval expertly, reclassifying them as experienced virgins (Carcea et al., 2021; Dvorkin & Shea, 2022; Ehret et al., 1987; Koch & Ehret, 1989; Marlin et al., 2015; Nowlan et al., 2024; Rosenblatt, 1967; Schiavo et al., 2020). With this shift in pup-directed behavior following maternal experience comes a shift in the neural representation of pup calls, particularly in the left primary auditory cortex (A1). Both mothers and experienced virgins, but not naïve virgins, demonstrate pup call-evoked, time-locked neural activity in left A1 (Liu et al., 2006; Liu & Schreiner, 2007; Marlin et al., 2015; Rothschild et al., 2013). This experience-dependent neural plasticity in left A1 is often referred to as maternal plasticity, and it has been shown to be facilitated by the neuropeptide oxytocin (OXT). OXT acts on left A1 by rebalancing excitatory and inhibitory responses to pup calls, thus enhancing pup call-evoked spiking and enabling adaptive maternal care (Marlin et al., 2015; Schiavo et al., 2020).

It remains unknown whether, alongside this auditory maternal plasticity, other brain regions exhibit similar changes in their pup call responses, leaving our understanding of the maternal brain incomplete. While some studies have reported experience-dependent responses to pup calls in select higher-order auditory areas (Fichtel & Ehret, 1999; Tasaka et al., 2020), no study to date has surveyed whether such responses coincide with alternations throughout the entire mouse brain. Given the complexity of pup call-evoked behaviors that maternally experienced mice exhibit, we hypothesized that pup calls engage neural circuity beyond auditory areas, in a manner that reflects differences in maternal experience among female mice.

In this study, we utilized refined behavioral assays and whole-brain activity mapping via tissue clearing and c-Fos staining to evaluate how pup call behavioral and neural responses change with maternal experience. By examining naïve virgins, experienced virgins, and mothers, we sought to disentangle innate and learned aspects of maternal plasticity. Our findings revealed unexpected behavioral differences between experienced virgins and mothers, as well as differences in the coordinated activity of brain regions upon pup call presentation across all three groups that may serve to allow for more efficient maternal responses to infant distress cues in experienced animals.

## Pup retrieval and pup call preference change with maternal experience

To characterize how maternal experience arising from either innate or learned experience affects behavioral and neural responses to pup calls, we studied three groups: naïve virgin females (no maternal behavior), experienced virgin females (learned maternal behavior), and mothers (innate maternal behavior). Naïve virgins were female mice who have had no experience caring for pups. Experienced virgins were female mice who initially had no experience caring for pups, then were cohoused with a mother and her newborn litter for three to six days – a technique shown to initiate maternal behaviors in females via social learning (Carcea et al., 2021; Dvorkin & Shea, 2022; Ehret et al., 1987; Koch & Ehret, 1989; Marlin et al., 2015; Nowlan et al., 2024; Rosenblatt, 1967; Schiavo et al., 2020). Mothers were non-pregnant female mice who had successfully weaned two litters, with the second litter weaned one to four weeks prior to experiments.

**Extended Figure 1:**
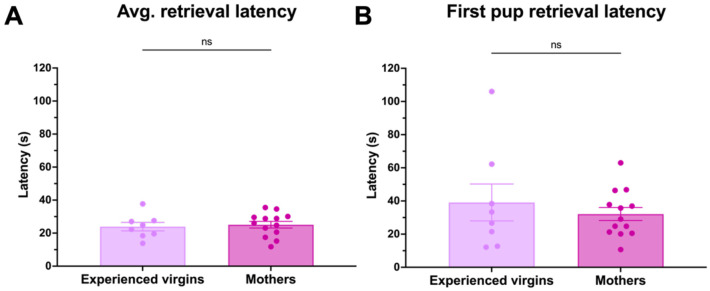
Mothers and experienced virgins exhibit indistinguishable pup retrieval latency. (A) Average pup retrieval latency per mouse. Unpaired t-test revealed no group effect (p = 0.7181; n = 8 experienced virgins, 13 mothers). Error bars denote mean ± SEM. (B) First trial pup retrieval latency per mouse. Unpaired t-test revealed no group effect (p = 0.4914; n = 8 experienced virgins, 13 mothers). Error bars denote mean ± SEM.

**Figure 1:**
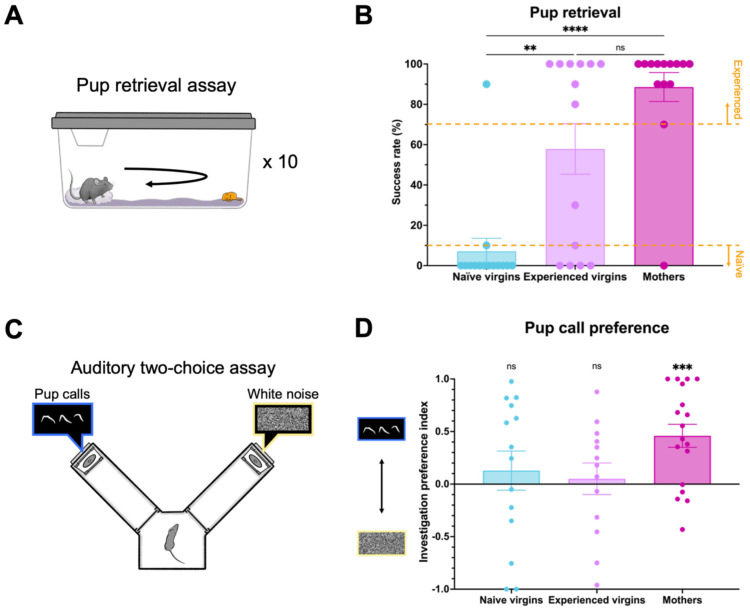
Mothers and experienced virgins retrieve pups successfully, but only mothers prefer pup calls. (A) Pup retrieval assay protocol, see Methods for details. (B) Pup retrieval success per mouse. Kruskal-Wallis test revealed significant group effect (p < 0.0001). Comparisons on graph represent Tukey’s post-hoc tests (naïve virgins vs. experienced virgins: p = 0.0097; naïve virgins vs. mothers: p < 0.0001; experienced virgins vs. mothers: p = 0.4431; n = 14 per group). Error bars denote mean ± SEM. **** p < 0.0001, ** p < 0.01. (C) Auditory 2-choice assay protocol (pup calls vs. white noise), see Methods for details. (D) Pup call investigation preference index per mouse during first 2 minutes of trial, see Methods for details. 1-sample t-tests (versus 0) revealed only mothers prefer pup calls (naïve virgins: p = 0.5028; experienced virgins: p = 0.7424; mothers: p = 0.0006; n = 14 naïve virgins, 13 experienced virgins, 18 mothers). 1-way ANOVA revealed no significant group effect (p = 0.2674; n = 14 naïve virgins, 13 experienced virgins, 18 mothers). Error bars denote mean ± SEM. Comparisons on graph represent 1-sample t-tests. *** p < 0.001.

To establish these experimental groups, each mouse underwent a pup retrieval assay (Figure 1A, Supplemental Video 1). To be considered naïve, virgins had to retrieve fewer than 10% of pups (thus screening out spontaneous retrievers); to be considered experienced, post-cohousing virgins and mothers had to retrieve at least 70% of pups (to only include experienced mice that retrieve pups reliably). In addition to screening mice prior to experiments, the pup retrieval assay also served to eliminate novelty as a potential confound in experimental results, as each mouse was exposed to pups and pup calls through this procedure. Our results showed that experienced virgins and mothers exhibited similar pup retrieval success (Figure 1B), and experienced virgins and mothers also exhibited indistinguishable pup retrieval latency (Extended Figure 1), highlighting the successful nature of pup-retrieval in the maternally experienced groups.

Pup calls are crucial for eliciting the act of pup retrieval (Hernandez-Miranda et al., 2017b; Schiavo et al., 2020). Prior work has shown that the presentation of pup calls is sufficient to attract mothers, but not naïve virgins, to a speaker (Ehret, 1987; Ehret et al., 1987; Schiavo et al., 2020; Sewell, 1970; Tasaka et al., 2020). This maternal attraction towards pup calls has been demonstrated in various studies through two-choice assays, in which competing speakers in an arena play different auditory stimuli at the same time, and the behavior of a mouse in that arena is quantified. One limitation of prior studies that used two-choice assays to assess pup call preference, however, is that pup odors were introduced into the arena prior to testing via pup retrieval or the presence of pups in the nest. This presents a confound, as prior research has shown that the presentation of pup odors amplifies A1 neural responses to pup calls in experienced virgins and mothers (Cohen et al., 2011; Cohen & Mizrahi, 2015).

**Extended Figure 2:**
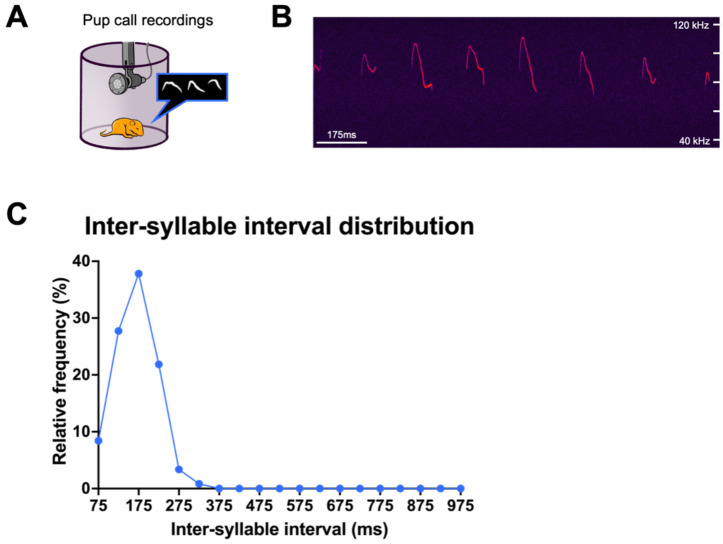
Pup call isolation and analysis. (A) Pup call recording protocol, see Methods for details. (B) Representative excerpt from pup call stimulus file, see Methods for details. (C) Frequency distribution of inter-syllable intervals in pup call stimulus file (n = 188). Dots indicate bin center (± 25 ms).

**Extended Figure 3:**
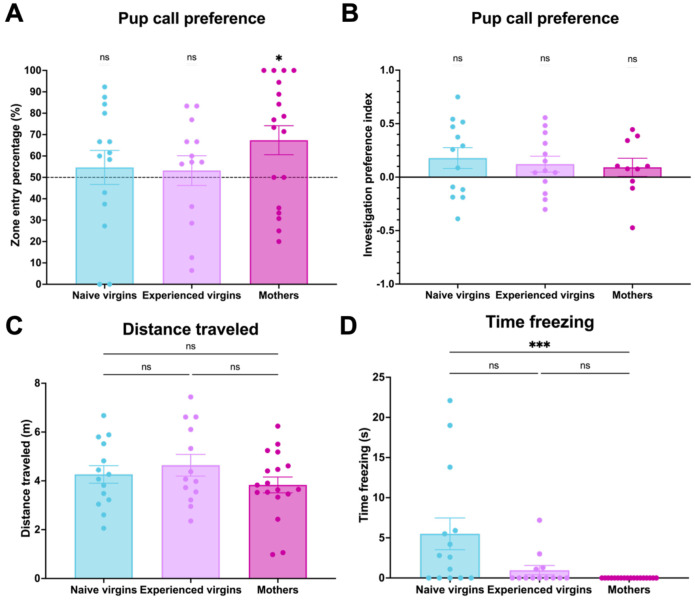
Additional auditory 2-choice assay behavioral measures. (A) Pup call zone entry percentage per mouse during first 2 minutes of trial. 1-sample Wilcoxon tests (versus 50%) revealed only mothers prefer pup calls (naïve virgins: p = 0.4915; experienced virgins: p = 0.6257; mothers: p = 0.0127; n = 14 naïve virgins, 13 experienced virgins, 18 mothers). Kruskal-Wallis revealed no significant group effect (p = 0.2674; n = 14 naïve virgins, 13 experienced virgins, 18 mothers). Dashed line represents no preference (50%). Error bars denote mean ± SEM. Comparisons on graph represent 1-sample Wilcoxon tests. * p < 0.05. (B) Investigation preference index per mouse during full 10 minutes of trial. 1-sample t-tests revealed no preferences (naïve virgins: p = 0.0919; experienced virgins: p = 0.1287; mothers: p = 0.3083; n = 13 naïve virgins, 13 experienced virgins, 10 mothers). 1-way ANOVA revealed no significant group effect (p = 0.7794; n = 13 naïve virgins, 13 experienced virgins, 10 mothers). Error bars denote mean ± SEM. Comparisons on graph represent 1-sample t-tests. (C) Distance traveled during first 2 minutes of trial. 1-way ANOVA revealed no significant group effect (p = 0.3101; n = 14 naïve virgins, 13 experienced virgins, 18 mothers). Error bars denote mean ± SEM. (D) Time spent freezing (threshold: at least 1 second) during first 2 minutes of trial. Kruskal-Wallis test revealed significant group effect (overall: p = 0.0003; naïve virgins vs. experienced virgins: p = 0.0977; naïve virgins vs. mothers: p = 0.0002; experienced virgins vs. mothers: p = 0.2915; n = 14 naïve virgins, 13 experienced virgins, 18 mothers). Error bars denote mean ± SEM. *** p < 0.001.

To investigate preference towards pup calls in the absence of other multisensory pup cues, we recorded pup calls from female and male six-day-old pups. We then confirmed that the temporal and spectral features of these pup calls aligned with those reported in prior literature (Fonseca et al., 2021; Schiavo et al., 2020), and created auditory stimulus files (Extended Figure 2). We then designed an auditory two-choice assay that explicitly did not involve the placement of pups nor bedding in the arena at any point, eliminating pup olfactory cues (Figure 1C, Supplemental Videos 2-4). Given two minutes to freely explore a custom-built two-choice arena while competing auditory stimuli played, mothers had a significant preference towards interacting with a speaker playing pup calls, compared to a speaker playing white noise, while naïve virgins did not. Surprisingly, however, experienced virgins also showed no significant preference to pup calls, suggesting that while both mothers and experienced virgins retrieve pups, the neural circuitry supporting this behavior may differ (Figure 1D). These results held true whether preference was calculated based on time spent investigating the speakers (Figure 1D) or on the number of times the speaker investigation zones were entered (Extended Figure 3A), as prior studies have analyzed (Ehret, 1987; Ehret et al., 1987; Lin et al., 2013a; Schiavo et al., 2020; Sewell, 1970; Tasaka et al., 2020). If the assay was extended to 10 minutes, no group had a sustained preference (Extended Figure 3B). Preference during the first two minutes could not be explained by group differences in distance traveled (Extended Figure 3C), though it should be noted that naïve virgins froze significantly more than mothers, perhaps indicating that mothers were more active in their investigation behavior (Extended Figure 3D).

## Maternal experience alters the brain-wide representation of pup calls

Based on our findings from the pup retrieval and auditory two-choice assays, as well as prior work, we hypothesized that pup calls engage different neural circuitry in females with different maternal experience. We suspected that by investigating pup call responses beyond auditory areas, we could begin to better explain the unexpected difference in pup call preference we observed between experienced virgins and mothers. To do so, we measured pup call-evoked activity across the brain by using intact tissue clearing and activity mapping via c-Fos, a protein marker for neuronal activity.

**Extended Figure 4:**
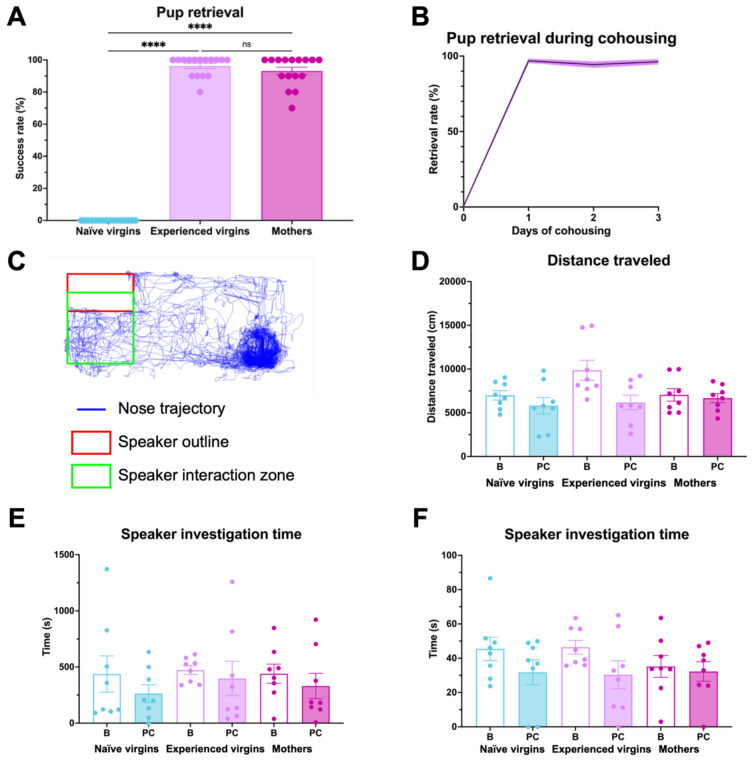
Behavior of mice included in whole-brain imaging data. (A) Pup retrieval success per mouse. Kruskal-Wallis test revealed significant group effect (p < 0.0001). Comparisons on graph represent Tukey’s post-hoc tests (naïve virgins vs. experienced virgins: p < 0.0001; naïve virgins vs. mothers: p < 0.0001; experienced virgins vs. mothers: p > 0.9999; n = 16 per group). Error bars denote mean ± SEM. **** p < 0.0001, ** p < 0.01. (B) Pup retrieval success of virgin females during cohousing with mother and litter (n = 16 experienced virgins). Purple shading denotes mean ± SEM. (C) Representative nose trajectory of a mouse during 30-minute sound exposure period, with speaker location (red) and interaction zone (green) outlined, see Methods for details. (D) Distance traveled per mouse during 30-minute sound exposure period (B = baseline, PC = pup calls). 2-way ANOVA revealed significant stimulus effect (group: p = 0.1330; stimulus: p = 0.0115; interaction: p = 0.1160; n = 8 per condition). Error bars denote mean ± SEM. (E) Speaker investigation time per mouse during 30-minute sound exposure period. 2-way ANOVA revealed no significant effects (group: p = 0.7594; stimulus: p = 0.2065; interaction: p = 0.9052; n = 8 per condition). Error bars denote mean ± SEM. (F) Speaker investigation time per mouse during first 2 minutes of sound exposure period. 2-way ANOVA revealed significant stimulus effect (group: p = 0.7000; stimulus: p = 0.0464; interaction: p = 0.5663; n = 8 per condition). Error bars denote mean ± SEM.

**Extended Figure 5:**
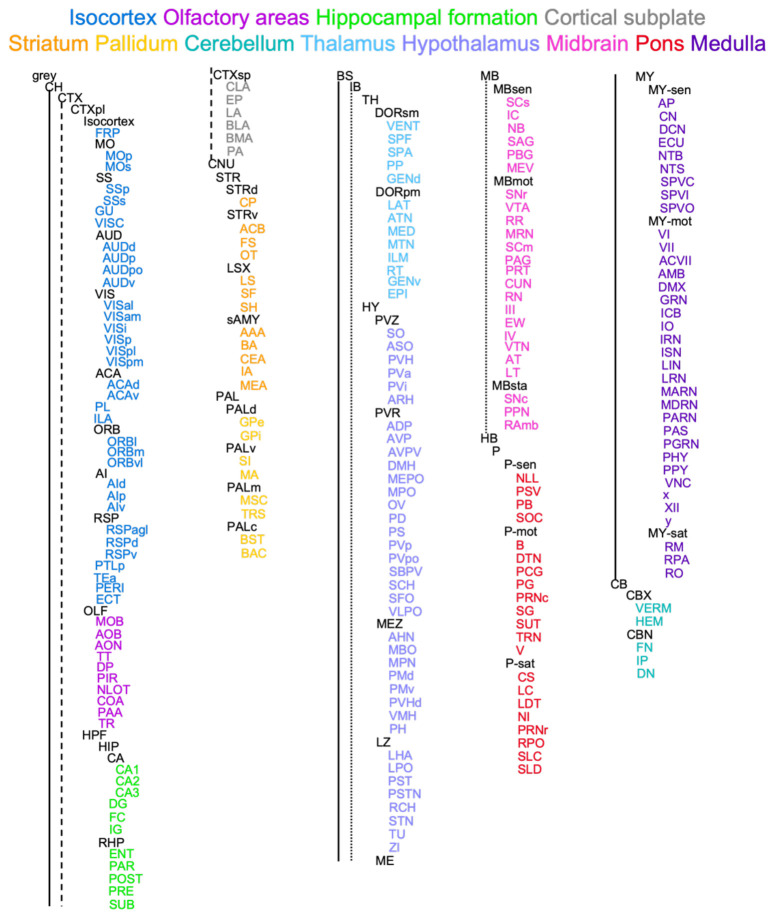
Partially collapsed Allen Brain Atlas. Rather than keeping the full 1,204 parcellations of the Allen Brain Atlas, the atlas was simplified down to 215 parcellations (see Table 1 for abbreviations). This was achieved by eliminating nonsense regions (i.e., ventricles, fibers, unlabeled) and limiting the level of specificity (i.e., layers). The 215 retained parcellations are represented as leaves in this tree, color-coded by broader brain area membership as indicated by the key at the top. Regions written in black text indicate that they were not included in the final 215.

**Extended Figure 6:**
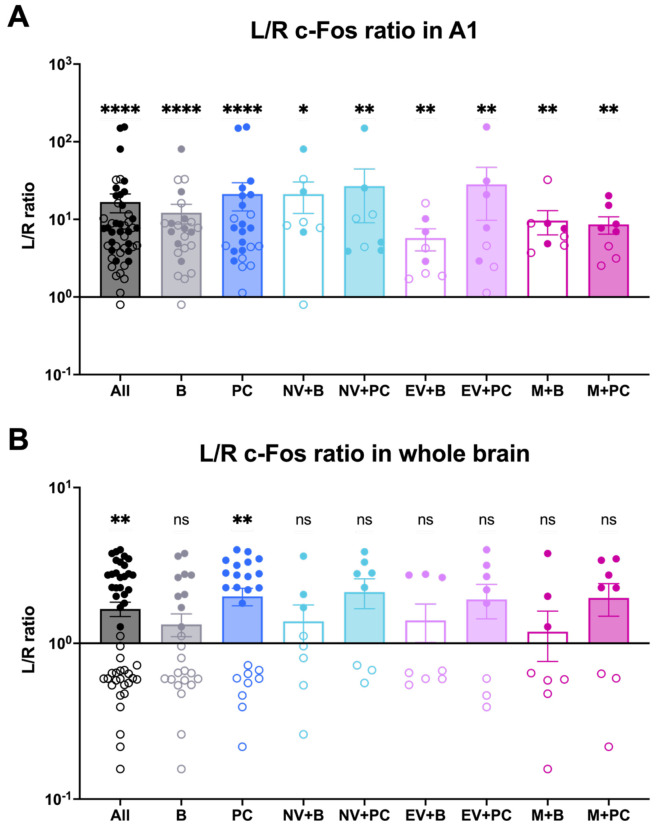
Lateralization in c-Fos dataset. Ratio (left/right) of number of c-Fos+ cells detected in A1 (A) or whole brain (B) of each sample, split by condition (All = all groups, all stimuli; B = all groups, baseline only; PC = all groups, pup calls only; NV+B = naïve virgins only, baseline only; NV+PC = naïve virgins only, pup calls only; EV+B = experienced virgins only, baseline only; EV+PC = experienced virgins only, pup calls only; M+B = mothers only, baseline only; M+PC = mothers only, pup calls only). Unfilled dots represent samples for which right hemisphere was imaged first. Error bars denote mean ± SEM. (A) Wilcoxon tests (versus 1) revealed significant lateralization in A1, regardless of condition (All: p < 0.0001, n = 48; B: p < 0.0001, n = 24; PC: p < 0.0001, n = 24; NV+B: p = 0.0156, n = 8; NV+PC: p = 0.0078, n = 8; EV+B: p = 0.0078, n = 8; EV+PC: p = 0.0078, n = 8; M+B: p = 0.0078, n = 8; M+PC: p = 0.0078, n = 8). **** p < 0.0001, ** p < 0.01, * p < 0.05. (B) Wilcoxon tests (versus 1) revealed minimal lateralization in whole brain, regardless of condition (All: p = 0.0059, n = 48; B: p = 0.6231, n = 24; PC: p = 0.0018, n = 24; NV+B: p = 0.6406, n = 8; NV+PC: p = 0.1094, n = 8; EV+B: p = 0.7422, n = 8; EV+PC: p = 0.1094, n = 8; M+B: p = 0.8438, n = 8; M+PC: p = 0.1094, n = 8). ** p < 0.01.

**Figure 2:**
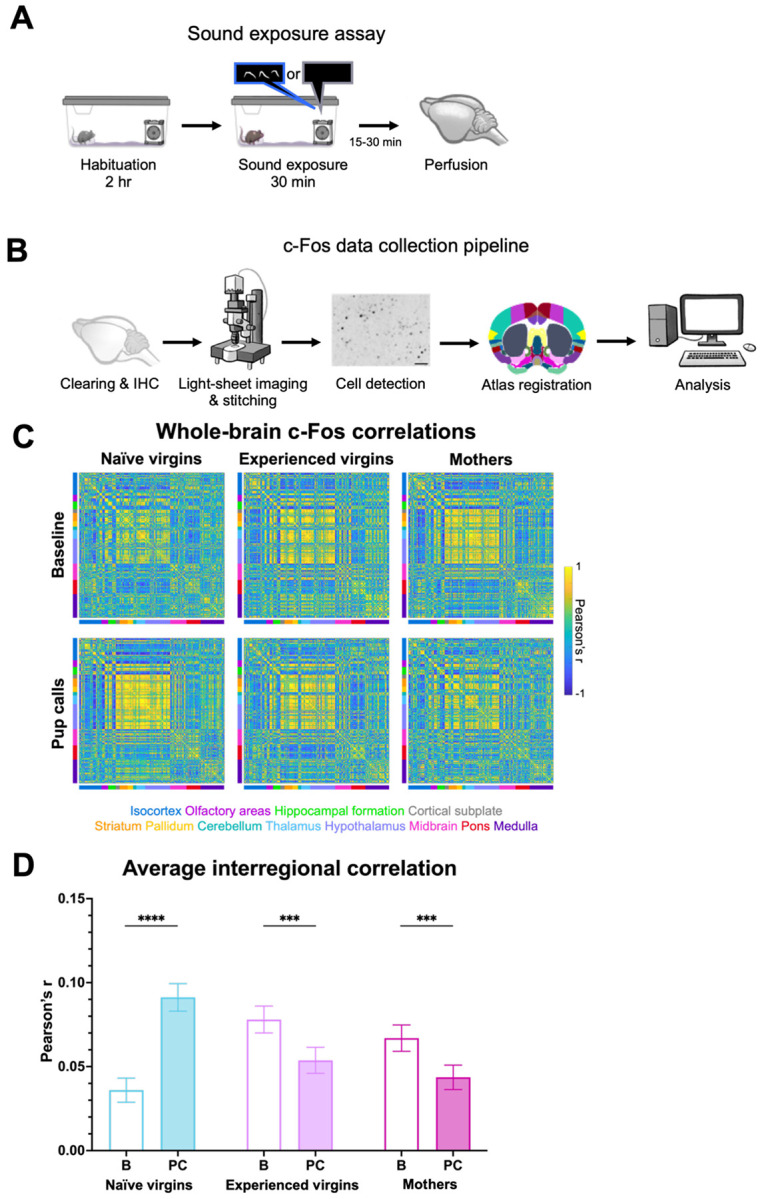
Pup calls exert an experience-dependent effect on brain-wide synchrony. (A) Sound exposure assay protocol, in which mice are presented with either pup calls or baseline (no stimulus), see Methods for details. (B) c-Fos data collection pipeline, which included iDISCO+ clearing, anti-c-Fos immunohistochemistry (IHC), light-sheet imaging and tile stitching, c-Fos+ cell detection via ClearMap, Allen Brain Atlas registration, and analysis (see Methods for details). Scale bar in representative image represents 25μm. (C) Pearson correlation matrices indicating interregional correlations, based on normalized c-Fos expression in baseline samples (top) and pup call-exposed samples (bottom) across groups (n = 8 x 215 regions). Color-blocked bars on axes correspond to broader brain areas according to the Allen Brain Atlas, see text below matrices for color coding. Matrix pixel colors represent Pearson correlation coefficients, see scale to the right. (D) Mean interregional correlation per condition (B= baseline, PC = pup calls). 2-way ANOVA following Fisher r-to-z transformation revealed significant effects of stimulus (p = 0.02) and of group and stimulus interaction (p < 0.0001). Comparisons on graph represent Tukey’s post-hoc tests of within-group baseline vs. pup calls (naïve virgins: p < 0.0001; experienced virgins: p = 0.0001796; mothers: p = 0.0004070; n = 8 x 215 regions). See Table 3 for other relevant pairwise comparisons. Error bars denote mean ± 95% CI. *** p < 0.001. **** p < 0.0001.

To characterize brain-wide c-Fos expression following pup call presentation, we designed a sound exposure assay (Figure 2A). As described earlier, mice belonging to either the naïve virgin, experienced virgin, or mother group were established via pup retrieval and cohousing (Extended Figure 4A). For the purposes of this experiment, pup retrieval of cohoused virgins was tested each day to monitor any major differences in learning over time, of which we found none (Extended Figure 4B). Then, for the sound exposure assay, mice were exposed for 30 min to either the presentation of pup calls or a baseline condition (no stimulus presented, with the same ambient noise as the pup calls condition). 15–30 min later, their brains were perfused, cleared and stained for c-Fos via the iDISCO+ protocol (Renier et al., 2014), imaged with a light-sheet microscope, and reconstructed in 3D (Figure 2B, Supplemental Videos 5-6). We then used the ClearMap pipeline to detect c-Fos+ cells, identify their spatial coordinates within the sample, and align the sample to the Allen Brain Atlas to assign each c-Fos+ cell to a brain region (Figure 2B, Supplemental Video 7) (Renier et al., 2016). We simplified the Allen Brain Atlas into 215 regions that still tiled the whole brain but limited the granularity of parcellations (Extended Figure 5, Table 1). We also summed c-Fos+ cell counts per brain region bilaterally. Before doing so, however, we first examined whether there was any lateralization in c-Fos expression. As expected based on prior literature (Ehret, 1987; Levy et al., 2019; Marlin et al., 2015), we observed higher c-Fos expression in the left A1 of pup call-exposed samples; surprisingly, however, c-Fos expression in baseline samples was also left lateralized in A1, across all groups (Extended Figure 6A). It should be noted that hemisphere imaging order affected c-Fos+ cell counts due to bleaching near the midline after the first imaging session, yet still left-lateralization in A1 prevailed. However, aside from the effect of hemisphere imaging order, we did not see the same lateralization across any conditions when comparing across the whole brain, justifying the bilateral summation of c-Fos+ cell counts within each brain region (Extended Figure 6B).

To assess any potential sources of movement confounds in our c-Fos data, we analyzed the behavior of mice during the sound exposure assay. We tracked the mice with DeepLabCut, a deep learning-based pose estimation pipeline (Mathis et al., 2018). We used tracking of the nose to assess distance traveled and time spent in the defined speaker investigation zone during sound exposure (Extended Figure 4C). Our behavioral analysis results revealed no significant group effects on distance traveled nor speaker investigation time during the 30-minute trial, indicating that no correction for movement was required for group-level c-Fos expression analyses (Extended Figure 4D-E). However, there was a significant effect of stimulus (pup calls versus baseline) on distance traveled during the 30-minute trial (Extended Figure 4D), as well as on speaker investigation time when analysis was restricted to the first two minutes of the trial (Extended Figure 4F), both indicating successful stimulus delivery and perception.

**Extended Figure 7:**
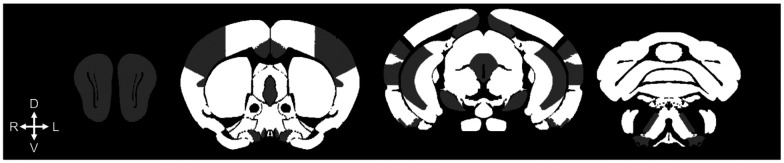
Randomly distributed batch effect. 4 representative coronal slices representing brain regions with (white) and without (gray) batch effect, as determined by 1-way ANOVA that used batch to explain normalized c-Fos per brain region (see Methods). Note the seemingly random spatial distribution that did not appear to have any directionality or clustering (D = dorsal, V = ventral, R = right, L = left).

Inspired by previous studies that analyzed similar datasets, we modeled c-Fos+ cell counts per region using a generalized linear mixed model (GLMM; see Methods) (Zimmerman et al., 2024). This analysis yielded 10 regions for which the inclusion of group and stimulus information provided better explanatory power, but only two regions passed correction for multiple comparisons (Table 2). It should be noted that compared to prior work using GLMMs to model brain-wide c-Fos, we were poorly powered, perhaps limiting the conclusions that can be drawn from this analysis regarding differences in regional c-Fos expression amplitude. We also observed a strong batch effect, which is a technical challenge for whole-brain immunohistochemistry. To address batch effects, we normalized c-Fos+ cell counts per region by total counts per sample, and we ensured that all conditions (all six different combinations of group and stimulus type) were equally represented per batch. Additionally, close examination of the spatial distribution of brain regions with significant batch effects after normalization revealed no apparent spatial pattern across the brain (Extended Figure 7). Given that our batches were balanced and batch effects were spread seemingly randomly throughout the brain, we moved forward with an analysis method that is robust to batch effect: correlation of normalized regional c-Fos expression across samples within each condition (Jin et al., 2025; Rogers et al., 2026; Wheeler et al., 2013).

To test whether pup calls engage different neural circuitry in females of different maternal experience, we correlated regional, normalized c-Fos expression across samples within our six conditions, producing six correlation matrices that represented interregional c-Fos correlations across the whole brain (Figure 2C). To quantify differences in brain-wide synchrony across conditions, we computed the average interregional correlation per matrix (Figure 2D). We found a significant effect of stimulus, as well as a significant interaction effect of group and stimulus, on average interregional correlation. This result demonstrated that pup calls (compared to baseline) had a significant effect on average interregional correlation across the whole brain, but that this effect differed per group. Indeed, compared to baseline, pup calls led to an increase in average brain-wide correlation in naïve virgins, while in the two maternally experienced groups, pup calls led to a decrease (Figure 2D, Table 3). Overall, when naïve animals perceive pup calls, we observe an increase in global synchrony. However, when experienced animals perceive pup calls, we observe a decrease in global synchrony, suggesting more localized responses in the maternally experienced groups.

**Extended Figure 8:**
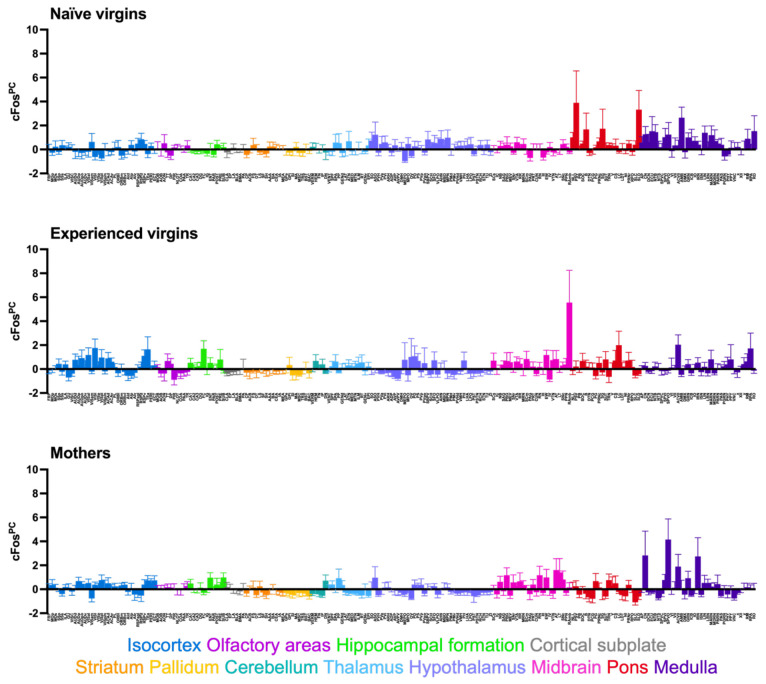
Baseline-standardized c-Fos expression. ***cFos^PC^*** per brain region (215 regions; see Table 1 for abbreviations), averaged per group (n = 8). See Methods for details. Error bars denote mean ± SEM.

**Extended Figure 9:**
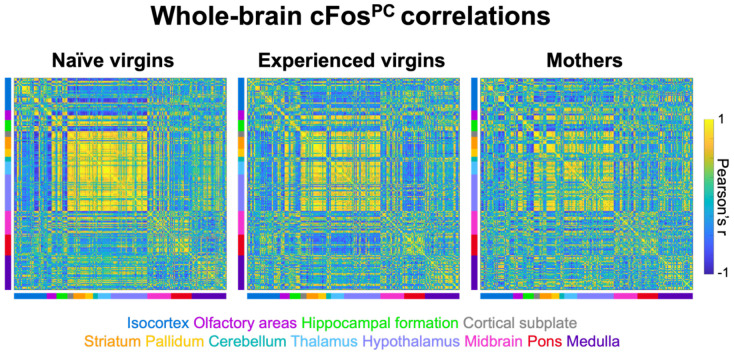
Brain-wide coordinated activation upon pup call exposure differs between groups. Pearson correlation matrices indicating interregional correlations, based on ***cFos^PC^*** in pup call-exposed samples across groups (n = 8 x 215 regions). Color-blocked bars on axes correspond to broader brain areas according to the Allen Brain Atlas, see text below matrices for color coding. Matrix pixel colors represent Pearson correlation coefficients, see scale to the right.

In addition to identifying an experience-dependent effect of pup calls on brain-wide coordination, our analysis also revealed that our three groups had significant differences in their correlated c-Fos expression at baseline (Table 3). To account for this fact and simplify further analyses, similar to how prior work has done (Jin et al., 2024; Rogers et al., 2026), we standardized each brain region’s pup call-exposed c-Fos expression to baseline expression within each group, calculating a measure we defined as *cFos^PC^* per region (see Methods). As a result, *cFos^PC^* represents each region’s c-Fos expression that we could attribute to pup call perception, accounting for baseline differences between naïve virgins, experienced virgins, and mothers (Extended Figure 8, Supplemental Videos 8-10). We then correlated regional *cFos^PC^* per group to measure the coordinated recruitment of brain regions during pup call perception, relative to baseline (Extended Figure 9). It should be noted that since the definition of *cFos^PC^* is mathematically equivalent to Z-scoring the pup call data based on within-group baseline data, thus altering the mean but not the relationship between pup call-exposed c-Fos data points, the *cFos^PC^* correlation matrices (Extended Figure 9) were identical to the pup call-exposed correlation matrices before baseline standardization (Figure 2C). Therefore, all further group comparisons were focused on *cFos^PC^* correlation analyses.

## Recruitment of Pup Avoidance and Parental Care Circuits upon pup call exposure reveal group differences

**Extended Figure 10:**
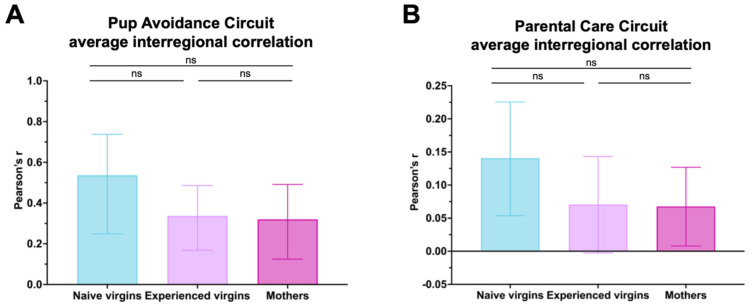
Pup Avoidance and Parental Care Circuits reveal similar synchrony between groups. (A) Mean interregional correlation in Pup Avoidance Circuit per group. Kruskal-Wallis test following Fisher r-to-z transformation revealed no significant group effect (p = 0.1752; n = 8 x 9 regions). Error bars denote mean ± 95% CI. (B) Mean interregional correlation in Parental Care Circuit per group. Kruskal-Wallis test following Fisher r-to-z transformation revealed no significant group effect (p = 0.6972; n = 8 x 27 regions). Error bars denote mean ± 95% CI.

**Figure 3:**
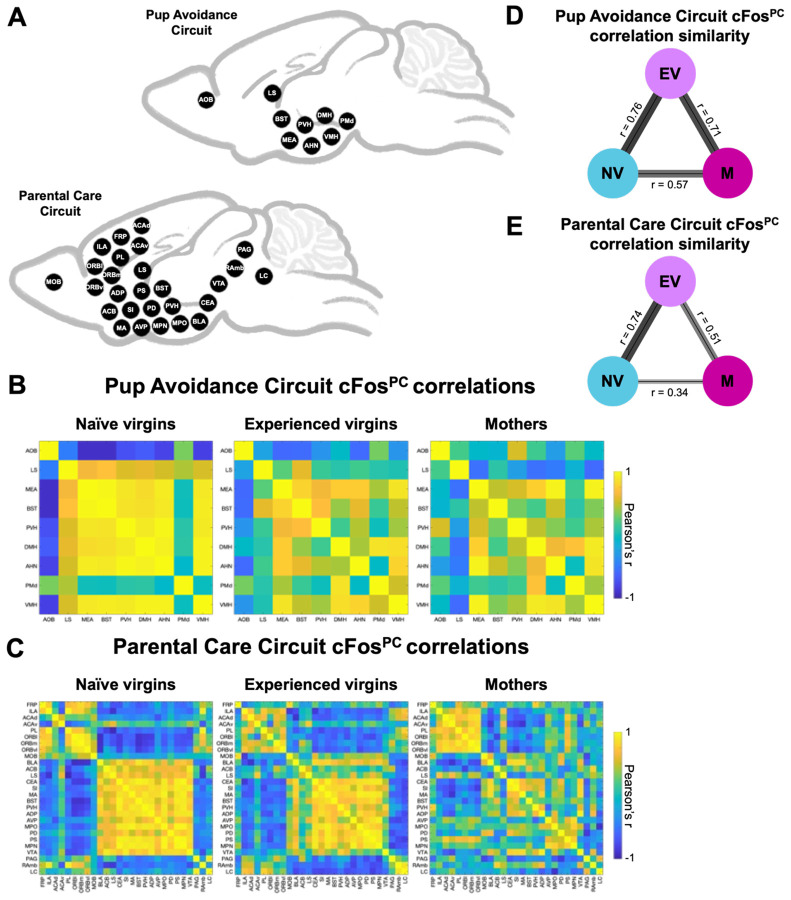
Pup Avoidance and Parental Care Circuits reveal different coordinated activation patterns upon pup call exposure between groups. (A) Regions within the Pup Avoidance and Parental Care Circuits, adapted from Dulac et al., 2014 (see Table 1 for abbreviations). (B) Pearson correlation matrices indicating interregional correlations in Pup Avoidance Circuit, based on ***cFos^PC^*** in pup call-exposed samples across groups (n = 8 x 9 regions). Matrix pixel colors represent Pearson correlation coefficients, see scale to the right. (C) Pearson correlation matrices indicating interregional correlations in Parental Care Circuit, based on ***cFos^PC^*** in pup call-exposed samples across groups (n = 8 x 27 regions). Matrix pixel colors represent Pearson correlation coefficients, see scale to the right. (D) Graphical representation of Pup Avoidance Circuit matrix similarity between groups (NV = naïve virgins, EV = experienced virgins, M = mothers), determined via Mantel test (naïve virgins vs. experienced virgins: p < 0.0001; naïve virgins vs. mothers: p = 0.000412; experienced virgins vs. mothers: p < 0.0001; n = 8 x 9 regions). Edge thickness and transparency are proportional to r value. (E) Graphical representation of Parental Care Circuit matrix similarity between groups, determined via Mantel test (naïve virgins vs. experienced virgins: p < 0.0001; naïve virgins vs. mothers: p < 0.0001; experienced virgins vs. mothers: p < 0.0001; n = 8 x 27 regions). Edge thickness and transparency are proportional to r value.

Foundational work in the field of maternal behavior across various species has led to the definition of two functionally opposing circuits that control (1) pup-directed avoidance or aggression (going forward, referred to as “pup avoidance”) and (2) parental behavior (Dulac et al., 2014) (Figure 3A). While these circuits were defined by drawing upon studies that largely examined multisensory pup interactions, we decided to use these circuits as a priori regions of interest. We correlated *cFos^PC^* across each subset of regions, allowing us to assess coordinated recruitment of these circuits upon pup call exposure in naïve virgins, experienced virgins, and mothers (Figure 3B-C). It is worth noting that we had to separate or combine some regions from the originally proposed circuits to map onto our level of Allen Brain Atlas parcellation specificity – for example, Dulac et al. refer to the prefrontal cortex (PFC), which we break down into the frontal pole (FRP), infralimbic area (ILA), dorsal and ventral anterior cingulate areas (ACAd, ACAv), prelimbic area (PL), and the lateral, medial, and ventrolateral orbital areas (ORBl, ORBm, ORBvl). To assess how these two circuits are similarly or differently recruited across groups, we first calculated the average interregional correlation to compare overall circuit synchrony, finding no significant group differences in either circuit (Extended Figure 10). However, the circuits appeared to have group-level differences in their spatial correlation patterns. To quantify and test this, we computed the similarity of these two circuits’ correlation matrices across groups (Figure 3D-E). In the Pup Avoidance Circuit, our results indicated that our three groups were relatively similar to each other, albeit with naïve virgins and mothers being the least similar pair (Figure 3D). In the Parental Care Circuit, however, there was more variation in the matrix similarities, with naïve virgins and experienced virgins being the most similar, experienced virgins and mothers being moderately similar, and naïve virgins and mothers being most dissimilar from each other (Figure 3E). Intriguingly, this gradient in similarity follows the gradient of maternal experience across groups. Furthermore, the fact that naïve virgins and experienced virgins showed the highest similarity in the Parental Care Circuit upon pup call exposure coincides with the surprising result of our auditory two-choice assay, which showed that, like naïve virgins, experienced virgins do not prefer pup calls, even though they do retrieve pups.

## Maternal experience leads to a more fine-tuned pup call response network

**Figure 4:**
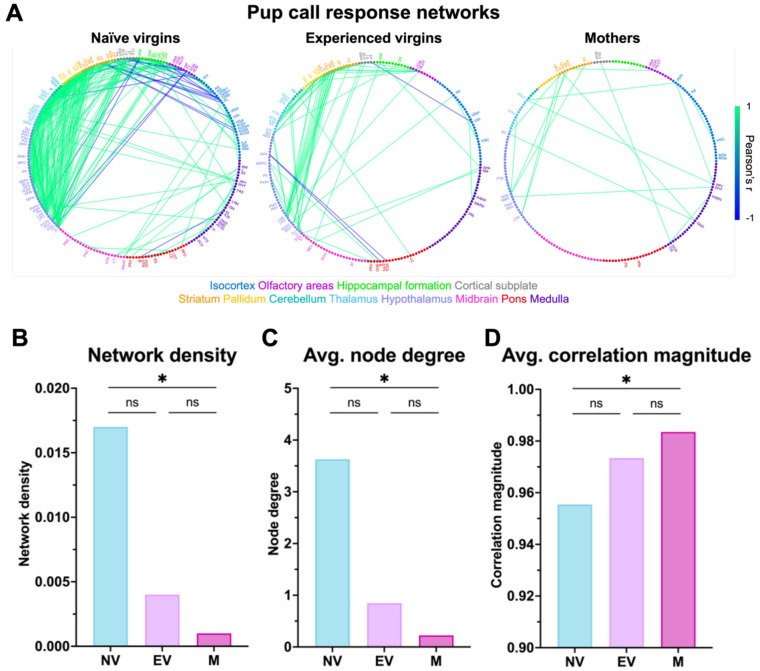
Maternal experience leads to sparser, but stronger, pup call response networks. (A) Pup call response networks, represented as undirected graphs, derived from ***cFos^PC^*** correlation matrices, thresholded at FDR < 0.05 and corrected for multiple comparisons via the Benjamini, Krieger and Yekutieli 2-stage step-up method. Nodes represent 215 brain regions, node marker colors correspond to broader brain areas according to the Allen Brain Atlas, see text below graphs for color coding. Only nodes that participate in edges are labeled (see Table 1 for abbreviations). Edge color represents Pearson correlation coefficients, see scale to the right. (B) Network density per group’s pup call response network (NV = naïve virgins, EV = experienced virgins, M = mothers). Comparisons on graph derived from permutation tests (naïve virgins vs. experienced virgins: p = 0.094; naïve virgins vs. mothers: p = 0.038; experienced virgins vs. mothers: p = 0.309; n = 8 x 215 regions; n = 1,000 permutations). Bars denote observed values. * p < 0.05. (C) Average unweighted node degree per group’s pup call response network. Comparisons on graph derived from permutation tests (naïve virgins vs. experienced virgins: p = 0.094; naïve virgins vs. mothers: p = 0.038; experienced virgins vs. mothers: p = 0.308; n = 8 x 215 regions; n = 1,000 permutations). Bars denote observed values. * p < 0.05. (D) Average correlation magnitude per each group’s pup call response network. Comparisons on graph derived from permutation tests (naïve virgins vs. experienced virgins: p = 0.098492; naïve virgins vs. mothers: p = 0.020325; experienced virgins vs. mothers: p = 0.2438; n = 8 x 215 regions; n= 1,000 permutations). Bars denote observed values. * p < 0.05.

To identify how maternal experience alters the brain-wide network of regions recruited during pup call perception, we applied stringent significance thresholding to our three groups’ brain-wide *cFos^PC^* correlation matrices (Extended Figure 9) and corrected for multiple comparisons. The surviving pairwise correlations were used to define pup call response networks, in which each node is one of our 215 brain regions, and each edge is a surviving *cFos^PC^* correlation between two regions (Figure 4A). Then, the network density, average node degree, and average correlation magnitude were calculated for the pup call response network of naïve virgins, experienced virgins, and mothers, and group-level differences were assessed via permutation testing (Figure 4B-D).

We found that naïve virgins had the highest pup call response network density, followed by experienced virgins, and then mothers (Figure 4B). The same pattern was revealed for average node degree (Figure 4C, Table 4), and the reverse pattern was found for average correlation magnitude (Figure 4D, Table 5). These three network measures once again identified gradients in the brain-wide representation of pup calls that parallel the gradient of maternal experience across groups. Permutation tests of these network measures confirmed that mothers had a significantly lower network density, lower average node degree, and higher average correlation magnitude, compared to naïve virgins, setting these two groups apart as the most distinct. Comparisons between naïve virgins versus experienced virgins, as well as between experienced virgins versus mothers, revealed trending though non-significant results, suggesting that experienced virgins represent an intermediate phenotype between naïve virgins and mothers that is more similar to mothers. Given that network density is particularly affected by the thresholding used to turn *cFos^PC^* correlation matrices into pup call response networks, we conducted a sensitivity analysis to confirm that our results held regardless of threshold choice, which we found to be true (Table 6). Taken together, these network-level analyses revealed that maternally experienced animals exhibit a sparser, yet stronger, pup call response network, compared to naïve animals. This finding reveals a more specialized brain-wide pup call response that is reflected in pup retrieval success in experienced animals.

**Extended Figure 11:**
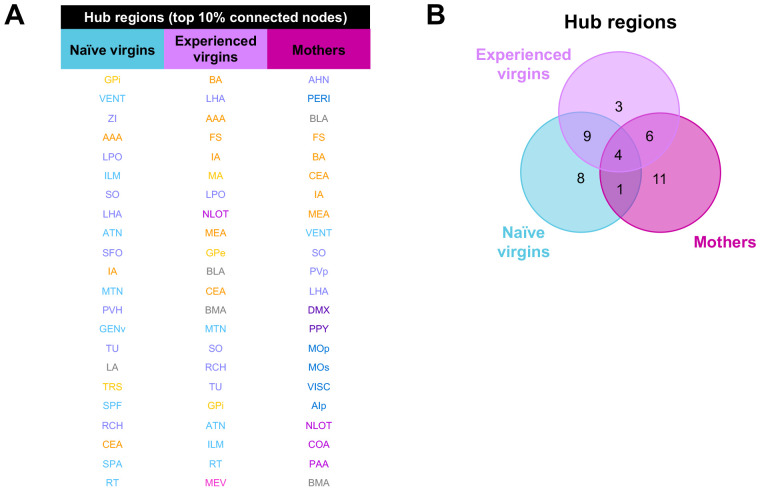
Pup call response hub regions across groups. (A) Pup call response hub regions (top 10% most connected nodes, as defined by node degree; see Table 4) per group. (B) Venn diagram depicting number of unique and overlapping hub regions.

**Figure 5:**
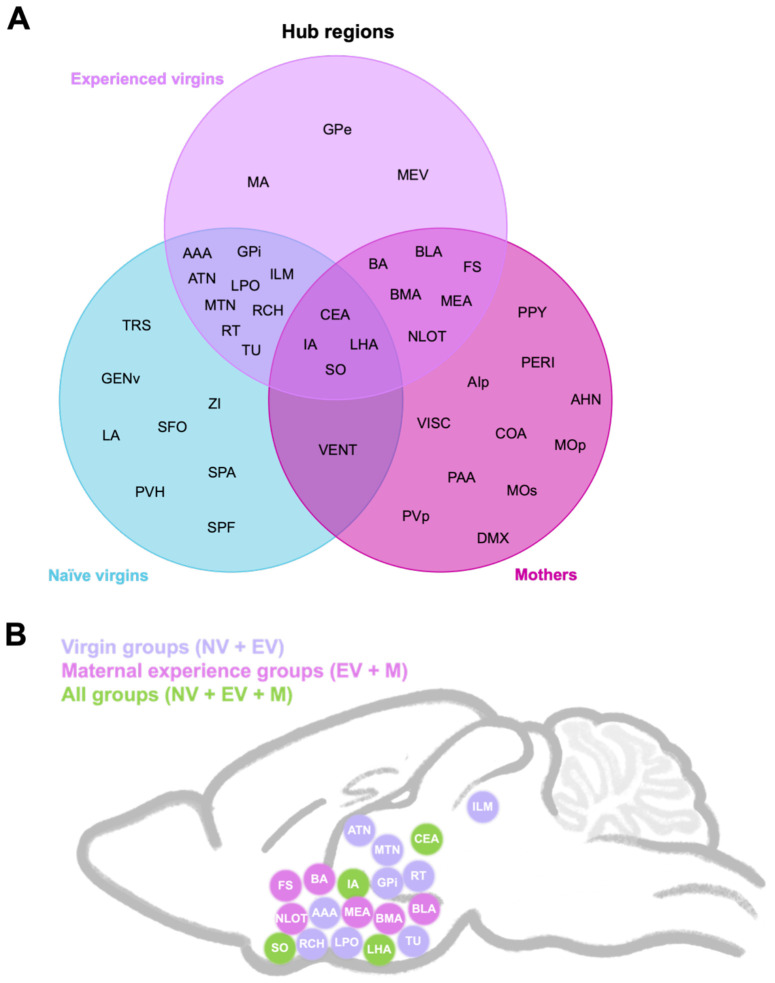
Pup call response hub regions across groups. (A) Venn diagram depicting abbreviations of unique and overlapping hub regions (hubs defined as top 10% most connected nodes per pup call response network) between groups (see Table 1 for region abbreviations). (B) Schematic of pup call response hub regions (purple = hub shared across virgin groups, naïve and experienced virgins; pink = hub shared across maternally experienced groups, experienced virgins and mothers; green = hub shared across all 3 groups).

To understand which brain regions may play a key role in coordinating each group’s brain-wide pup call response network, we extracted the top 10% of connected nodes, representing the hub regions in each group’s pup call response network (Figure 5A, Extended Figure 11A-B, Table 4). We then compared the sets of hub regions between groups (Figure 5A, Extended Figure 11B). This exploratory analysis revealed that there is minimal overlap in hub regions between groups, aside from four fully overlapping regions: the central amygdala (CEA), intercalated amygdalar nucleus (IA), lateral hypothalamic area (LHA), and supraoptic nucleus (SO) (Figure 5A-B). Other hub regions stand out as being shared between virgin groups (naïve and experienced virgins), or between maternally experienced groups (experienced virgins and mothers) (Figure 5B).

Overall, our pup call response network analyses revealed that pup calls recruit different networks in naïve virgins, experienced virgins, and mothers. Furthermore, mothers recruit a pup call response network that is sparser, yet stronger, than that of naïve virgins, with experienced virgins appearing to show an intermediate effect of maternal experience.

## Pup call responses in OXT projection-dense regions

Prior work revealed that maternal plasticity in left A1 is facilitated by OXT, which acts by rebalancing excitatory and inhibitory responses to pup calls to sensitize A1 to this behaviorally-relevant stimulus (Carcea et al., 2021; Marlin et al., 2015). OXT+ cells in the paraventricular nucleus of the hypothalamus (PVH) have been shown to respond to pup calls, receiving inputs from a non-canonical auditory pathway, allowing for pup call-evoked OXT release throughout the brain (Valtcheva et al., 2023). Furthermore, previous studies have shown that expression of the OXT receptor is particularly prevalent in inhibitory interneurons (K. Li et al., 2016; Marlin et al., 2015; Mitre et al., 2016; Nakajima et al., 2014; Schimmer et al., 2026), perhaps explaining why exogenous OXT injection was shown to have an overall inhibitory effect on brain activity (Choe et al., 2022). With this in mind, we hypothesized that pup calls recruit a sparser network in mothers due to brain-wide inhibition via OXT signaling.

**Figure 6:**
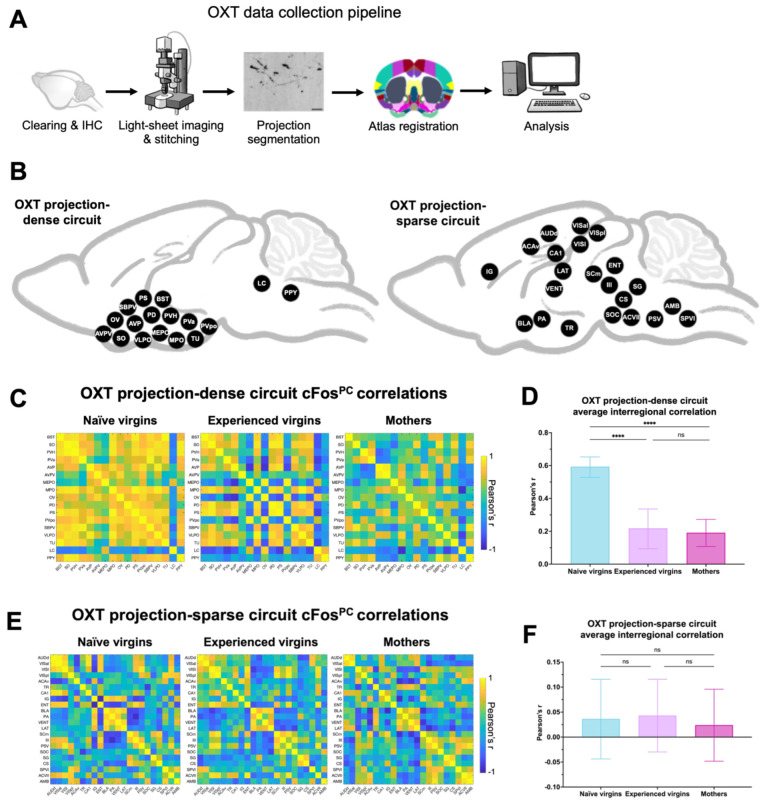
OXT projection-dense circuit reveals experience-dependent synchrony upon pup call exposure between groups. (A) OXT data collection pipeline, which included iDISCO+ clearing, anti-OXT immunohistochemistry (IHC), light-sheet imaging and tile stitching, OXT projection segmentation via our custom segmentation model, Allen Brain Atlas registration, and analysis (see Methods for details). Scale bar in representative image represents 25μm. (B) OXT projection-dense circuit (see Table 1 for abbreviations). (C) Pearson correlation matrices indicating interregional correlations in OXT projection-dense circuit, based on ***cFos^PC^*** in pup call-exposed samples across groups (n = 8 x 17 regions). Matrix pixel colors represent Pearson correlation coefficients, see scale to the right. (D) Mean interregional correlation among OXT projection-dense circuit regions per group. Kruskal-Wallis test following Fisher r-to-z transformation revealed significant group effect (p < 0.0001). Comparisons on graph represent Tukey’s post-hoc tests (naïve virgins vs. experienced virgins: p < 0.0001; naïve virgins vs. mothers: p < 0.0001; experienced virgins vs. mothers: p > 0.9999; n = 8 x 17 regions). Error bars denote mean ± 95% CI. **** p < 0.0001.

To probe whether OXT may be playing the same sensitization role in the whole brain as it does in A1, we mapped OXT projections across the entire brain in naïve virgins, experienced virgins, and mothers. Using an anti-OXT antibody, we stained for OXT+ cells and their projections in the same samples that underwent iDISCO+ clearing, c-Fos staining, and light-sheet imaging (Figure 6A, Supplemental Video 11) (Renier et al., 2014). We then built upon published methods (Friedmann et al., 2020; Gongwer et al., 2023) to develop a deep learning-based pipeline to automatically segment OXT projections, identify their coordinates within the sample, and align the sample to the Allen Brain Atlas – applying the same Elastix transformations generated from the ClearMap pipeline – to assign each OXT+ voxel to one of our 215 brain regions (Figure 6A). We focused our analyses on the left hemisphere, as prior studies have tested and found no lateralization of OXT projections (Marlin et al., 2015; Mitre et al., 2016). We modeled OXT+ voxel counts per region using a GLMM (see Methods) (Zimmerman et al., 2024). This analysis yielded 16 regions for which the inclusion of group information provided better explanatory power, but no regions passed correction for multiple comparisons (Table 7). Again, compared to prior work using GLMMs, we were poorly powered, perhaps limiting the conclusions that can be drawn from this analysis regarding differences in regional OXT expression. Furthermore, our light-sheet imaging parameters were primarily optimized for c-Fos expression, thus we cannot rule out the possibility that increased resolution may reveal more nuanced insights regarding OXT projections. Nevertheless, through this analysis we found no significant regional differences in OXT projection density between naïve virgins, experienced virgins, and mothers. Given that we found no group differences, we averaged relative OXT projection density (see Methods) per region across all animals (Table 8, Supplemental Video 12) and extracted the top 10% of OXT projection-dense regions. This allowed us to establish an OXT projection-dense circuit that is common across all groups studied (Figure 6B).

To assess coordinated pup call-evoked responses in regions that receive direct OXT input, we correlated *cFos^PC^* across the OXT projection-dense circuit per group and calculated the average interregional correlation (Figure 6C-D). We found a significant group effect that suggested that our two maternally experienced groups recruited this OXT-dense circuit similarly, mirroring our findings in the whole brain. Specifically, experienced virgins and mothers showed lower synchrony in this circuit, compared to naïve virgins, perhaps indicating that inhibitory signaling was at play in these regions during pup call perception in experienced mice. Notably, this group effect was not present when we correlated *cFos^PC^* across the OXT projection-sparse circuit (Figure 6B, E-F), suggesting that OXT signaling may drive the experience-dependent group differences we observed at the level of the whole brain.

## Discussion

Prior work has revealed that maternal care can be unlocked in different ways: through innate processes in the case of mothers, or through observational learning in the case of experienced virgins. Both experienced virgins and mothers ultimately prove to be successful pup retrievers and to have robust, reliable neural responses to pup calls in left A1 (Marlin et al., 2015). However, we hypothesized that there may be nuanced differences in their pup call responses, especially if we extend our focus beyond left A1 to the entire brain. Our findings demonstrate that the experience of providing maternal care leads to previously unidentified changes in pup call-evoked behavioral and neural responses.

By designing and conducting a two-choice behavioral assay that solely presents auditory stimuli, in the absence of other multisensory pup cues, we found surprising differences between our two maternally experienced groups. As expected based on prior literature (Ehret, 1987; Ehret et al., 1987; Lin et al., 2013a; Schiavo et al., 2020; Sewell, 1970; Tasaka et al., 2020), mothers exhibited a preference for investigating a speaker playing pup calls, compared to a speaker playing white noise, while naïve virgins did not. However, experienced virgins also did not show a preference toward pup calls. This result conflicted with prior expectation due to the fact that both experienced virgins and mothers retrieve pups and show strong A1 pup call responses. While both experienced virgins and mothers help pups in need of care, their difference in pup call preference suggests they may perform maternal behavior for different underlying motivations. It is also possible that experienced virgins require multisensory cues to respond appropriately to pup calls, such as the pup odors that were incorporated into prior studies of their pup call preference (Ehret et al., 1987; Lin et al., 2013b; Schiavo et al., 2020). This could be explained by the phenomenon that coincident pup odor presentation amplifies pup call responses in A1 in mice with maternal experience (Cohen et al., 2011; Cohen & Mizrahi, 2015).

To uncover potential explanations regarding this unexpected difference in pup call-evoked behavior, we took advantage of whole-brain activity mapping via c-Fos to explore how regions across the brain are cooperatively recruited during pup call perception. One way we approached analyzing this data was to start with a priori circuits of interest, namely (1) the Pup-Directed Avoidance and Aggression Circuit and (2) the Parental Care Circuit (Dulac et al., 2014). In comparing the coordinated recruitment of the Pup Avoidance Circuit during pup call exposure, we found that naïve virgins, experienced virgins, and mothers were relatively similar. This result was unsurprising, as none of these groups showed an aversion to pup calls in the two-choice assay, and none of the mice in this study were aggressive towards pups during the pup retrieval assay. In comparing the coordinated recruitment of the Parental Care Circuit during pup call exposure, however, we found more striking differences between groups. The pattern of pup call-evoked correlation was most similar between naïve virgins and experienced virgins, and the least similar between naïve virgins and mothers. Unlike the Pup Avoidance Circuit, the Parental Care Circuit includes higher-order processing areas related to emotional valence, reward, arousal, and decision-making. Therefore, this group difference in c-Fos correlation pattern in the higher-order processing of pup calls may facilitate mothers’ unique pup call preference. Being attracted to pup calls would serve to support adaptive maternal care, especially pup retrieval. It may even confer resiliency, allowing mothers to still be motivated to investigate the source of pup calls even in uncertain or risky environments. In fact, recent work has shown that activity in the prelimbic (PL) and infralimbic (ILA) areas, both part of the Parental Care Circuit, is crucial to successful pup retrieval under threatening conditions in both experienced virgins and mothers (Wu et al., 2025).

Expanding our focus to the entire brain, we found that pup call exposure led to experience-dependent changes in brain-wide activity. Specifically, we found that pup calls, compared to baseline, elicited an increase in average interregional correlation in naïve virgins, while in the two maternally experienced groups, pup calls led to a decrease. Average interregional correlation can be interpreted as a measure of global coupling, or brain-wide synchrony. While at first it may be perceived as counterintuitive, the concept that pup calls shift the experienced brain into a state of lower synchrony may suggest that the maternally experienced brain is more prepared to process this stimulus with less energetic demand. This idea would align with prior work in the field of the Neural Efficiency Hypothesis, which suggests that with expertise comes more efficient neural processing during a task (Bassett & Bullmore, 2017; Haier et al., 1988; L. Li & Smith, 2021; Neubauer & Fink, 2009). This notion was reinforced by our network-based analyses. We found that the pup call response network of mothers was more refined – sparser, yet stronger, compared to that of naïve virgins, and experienced virgins were positioned in between the two extremes.

By labeling and quantifying OXT projections across the whole brain, we identified a circuit of OXT projection-dense regions that were shared across naïve virgins, experienced virgins, and mothers. Looking specifically at pup call-evoked coordinated activity within this circuit, we found a maternal experience-dependent shift toward lower synchrony during pup call perception. This shift may be facilitated by OXT-mediated inhibition. Pup calls have been shown to activate OXT-expressing neurons in the paraventricular nucleus of the hypothalamus (PVH) of mothers, leading to a release in OXT (Valtcheva et al., 2023). Prior work, which also employed c-Fos-iDISCO+, showed that endogenous OXT release via PVH stimulation leads to less brain-wide c-Fos expression in wild-type mice (Choe et al., 2022). This brain-wide activity suppression may be achieved by OXT binding to OXT receptor-expressing cells, which have a high likelihood of being inhibitory interneurons (K. Li et al., 2016; Marlin et al., 2015; Mitre et al., 2016; Nakajima et al., 2014; Schimmer et al., 2026). Taken together, pup call-evoked OXT release could lead to brain-wide inhibition in OXT receptor-expressing cell populations, which in turn could lead to lower synchrony, both in our defined OXT projection-dense circuit and in the brain at large. However, it should be noted that pup calls are so far only known to induce OXT release in mothers, thus it is unclear if pup calls could have the same OXT-mediated, brain-wide effect in experienced virgins.

Our pup call response network results parallel nicely with recent human neuroimaging research. Recent studies have shown that the transition to motherhood is marked by a reduction in grey matter volume (GMV) that is most drastic around the time of parturition and has been associated with measures of mother-infant attachment. This GMV reduction only partially recovers, even after six years postpartum, and it is primarily only observed, at least to this degree, in gestational mothers and not their partners who are fathers or non-birthing mothers (Hoekzema et al., 2017; Martínez-García et al., 2021; Pawluski et al., 2022; Pritschet et al., 2024; Servin-Barthet et al., 2025). A current hypothesis in the field is that these GMV reductions indicate a period of maturation that supports the transition into motherhood, enhancing the cognitive abilities of new mothers in areas such as social and emotional processing – abilities that play a significant role in adaptive maternal caregiving (Anderson & Rutherford, 2012).

Taken together, the results presented in this study demonstrate that adaptive responses to pups and pup calls are associated with more refined pup call response networks that span the entire brain. These sparser, but stronger pup call response networks may allow maternally experienced mice to perceive and respond to pup calls more efficiently, supporting caregiving behavior.

## Materials and Methods:

### Mice

All procedures were approved by the Columbia University Institutional Animal Care and Use Committee. All mice in this project were from a C57BL/6J background, age-matched, and obtained from the Jackson Laboratory. All mice were housed with a 12 hour light/12 hour dark cycle, with food and water available ad libitum with the same food source supplied to all animals.

### Breeding and cohousing

During breeding, a virgin male and female were paired per cage and monitored regularly while they completed two rounds of breeding, two produce experienced mothers. Just before the birth of the second litter, the father was removed. The mothers studied were one to four weeks past the weaning of their second litter, to avoid capturing maternal separation effects in our experiments, and tested to ensure they retrieved pups reliably (at least 70% success in pup retrieval assay). The experienced virgins studied were first tested for baseline pup retrieval while they were naïve to pups, to ensure they were not spontaneous retrievers (no more than 10% success in pup retrieval assay) nor aggressive towards pups. Then, they were gently tail-marked with a permanent marker and placed in the home cage of a second-time mother with a newborn litter of age postnatal day one. If cohoused virgins were tested daily for pup retrieval, then they were tested every 24 hours until 72 hours after the start of cohousing. Otherwise, they were just tested at 72 hours. If the cohoused virgins met the criteria of successful retrieval (at least 70%) after 72 hours, then they were considered experienced. If they were not successful after 72 hours, they were placed back into cohousing and tested every 24 hours going forward until they met criteria. The naïve virgins studied were also tested for pup retrieval to ensure they were not spontaneous retrievers (no more than 10% success) nor aggressive towards pups.

### Pup retrieval assay

Pup retrieval tests were performed in a home cage with normal nesting material under normal white light, and they were video recorded from above. The test mouse was first allowed to habituate to the room for a minimum of two hours. To start a trial, one pup was placed in a corner of the cage, specifically in one of the two corners furthest from the nest (alternated per trial). The test mouse was then allotted two minutes. If within those two minutes, the test mouse retrieved the pup back to the nest, the trial was noted as a success. If those two minutes passed and the pup was still outside the nest, or if any pup-directed aggression was observed, the trial was deemed a failure. Each pup retrieval test consisted of 10 trials. Pups used for retrieval testing were generally of the age postnatal day four to six, except in the case of daily pup retrieval testing in cohoused virgins, in which case test pups could be as young as postnatal day one for baseline testing before cohousing.

### Pup retrieval assay behavioral analysis

Pup retrieval behavior was scored by blinded experimenters using BORIS (Behavioral Observation Research Interactive Software) (Friard & Gamba, 2016), and these observations were used to determine retrieval success per trial and retrieval latency. Average latency to retrieve per trial, latency to first retrieval, and overall retrieval success out of 10 trials was recorded per test mouse. The criteria used for pup retrieval included that naïve animals must exhibit 10% or lower success, otherwise they were considered spontaneous retrievers, and experienced animals must exhibit 70% or higher success. Experienced virgins and mothers were held to the same criteria.

### Pup call recordings

Pup isolation calls were recorded in a sound-isolated chamber. Male and female pups of age postnatal day six were individually placed on a clean Petri dish and placed in the recording chamber. An ultrasonic microphone (Avisoft Bioacoustics Condenser Ultrasound Microphone CM16/CMPA paired with UltraSoundGate 116H and controlled by Avisoft-RECORDER software) was hung from an opening in the center of the chamber ceiling and lowered to be around one inch above the pup. Following one minute of habituation, after which the pups usually began to vocalize, a recording of three to five minutes was acquired.

### Auditory stimulus files

To create pup call stimulus files without audible background noise, all pup call recordings were 15 kHz high-pass filtered. To maintain the natural bout structure of pup calls, bouts from male and female postnatal day six pups were cropped and interlaced into a stimulus file, with an inter-bout-interval of one second. To ensure that the stimuli represented typical pup isolation calls, the stimulus file was run through the VocalMat pipeline (Fonseca et al., 2021), and the distribution of inter-syllable-intervals was compared to that which is reported in existing literature (Schiavo et al., 2020). To create pup call versus white noise stimulus files for the two-choice assay, a pup call stimulus file and a zero to 100 kHz white noise file were matched in average volume and combined into a two-channel stimulus file.

### Auditory two-choice assay

Two-choice assays were performed in a custom-built, opaque white acrylic Y-shaped arena, with transparent acrylic lids and vertically removable partitions separating each chamber. Tests were video recorded from above. Ultrasonic speakers (Avisoft Bioacoustics Vifa Ultrasonic Dynamic Speaker, paired with UltraSoundGate Player 216H) were located outside of the arena, on the other side of a mesh opening in the arena wall. An ultrasonic microphone (Avisoft Bioacoustics CM16/CMPA Condenser Ultrasound Microphone, paired with UltraSoundGate 116H) was placed near one of the two speakers for confirmation of stimulus delivery. The test was split into two days: one habituation day and one test day. The habituation day began with one hour of habituation to the room in the home cage. Then, the test mouse was placed in the center chamber and allotted five minutes to explore the center chamber, followed by 10 minutes to freely explore the entire three-chamber arena after the doors were removed. The test day also began with one hour of habituation to the room in the home cage. Then, the test mouse was placed in the center chamber and allotted two minutes to explore the center chamber, followed by three minutes to freely explore the entire arena. Then, once the mouse was back in the center chamber, the doors were reinserted. Finally, the 10-minute stimulus file containing contrasting auditory stimuli (pup calls and white noise) was started just as the doors were removed once again, and the test mouse was allotted two to 10 minutes to freely explore the full arena during the playback of auditory stimuli. The speaker that played pup calls versus white noise was randomized per test mouse.

### Auditory two-choice assay behavioral analysis

Two-choice assay videos were flipped before analysis such that pup calls were always on the left. Test animals had to spend at least one second in the center chamber to be included in analysis, to ensure both auditory stimuli were sampled. Animal head position was tracked via the software ANY-maze (Stoelting Co.), and interaction zones were delineated around each speaker face. Time spent in each interaction zone, as well as zone entries, were recorded per test mouse. A pup call interaction preference index was calculated per mouse, following the formula:

$$\mathrm{Investigation}\;\mathrm{preference}\;\mathrm{index}\;=\frac{\left(\mathrm{time}\;\mathrm{in}\;\mathrm{pup}\;\mathrm{call}\;\mathrm{interaction}\;\mathrm{zone})-(\mathrm{time}\;\mathrm{in}\;\mathrm{white}\;\mathrm{noise}\;\mathrm{interaction}\;\mathrm{zone}\right)}{\left(\mathrm{time}\;\mathrm{in}\;\mathrm{pup}\;\mathrm{call}\;\mathrm{interaction}\;\mathrm{zone})+(\mathrm{time}\;\mathrm{in}\;\mathrm{white}\;\mathrm{noise}\;\mathrm{interaction}\;\mathrm{zone}\right)}$$

### Sound exposure assay

Sound exposure assays were performed in a clean home cage with a transparent acrylic lid, normal nesting material, and an ultrasonic speaker (Avisoft Bioacoustics Vifa Ultrasonic Dynamic Speaker, paired with UltraSoundGate Player 216H) under red light. Tests were video recorded from above. The test mouse was first allowed to habituate to the room and cage for two hours. To start a trial, the ultrasonic speaker was gently plugged in, and the experimenter began video recording and playing either a 30-minute pup call stimulus file or no stimulus file. Following 30 minutes of the sound exposure period, the test mouse was left unperturbed until intracardiac perfusion 15 to 30 minutes after the end of sound exposure, or 45 to 60 minutes after the start of sound exposure, following published protocols assessing pup call-evoked c-Fos expression (Fichtel & Ehret, 1999; Geissler et al., 2016).

### Sound exposure assay behavioral analysis

For tracking of the nose, left ear, right ear, midbody, and tailbase, we used DeepLabCut (version 3) (Mathis et al., 2018; Nath et al., 2019). We labeled 20 frames taken from each of the 48 sound exposure videos, with 95% used for training. We used a ResNet-50 based neural network with default parameters for 200 epochs. Following analysis, body part coordinates were then extracted and further processed using custom MATLAB code.

We used MATLAB (version 2022a) to analyze mouse behavior during the sound exposure period. We used tracking data of the nose to represent each mouse’s location in the cage. For each top-view sound exposure assay video, the speaker location was outlined, and the speaker interaction zone was defined as the rectangle with the same width of the speaker and the height of five centimeters. The interaction zone extended five centimeters from the center point of the speaker, to capture timepoints when mice were either on the ground in front of the face of the speaker or climbing the face of the speaker, thus defining a timepoint of speaker interaction.

### Tissue clearing and staining

Mice were deeply anesthetized with isoflurane and fixed with an intracardiac perfusion of 4% paraformaldehyde (PFA) in PBS with heparin. All harvested brains were post-fixed overnight at 4°C in 4% PFA in PBS. Samples were processed with the iDISCO+ tissue clearing and immunolabeling protocol, as described at http://www.idisco.info/ (Renier et al., 2014). Samples were incubated in primary and secondary antibody solutions for 10 days each. For c-Fos, the antibodies used were rat monoclonal anti-c-Fos (primary; 1:1500; Synaptic Systems #226-017) and goat anti-rat 647 (secondary; 1:1000; ThermoFisher #A-21247). For oxytocin (OXT), the antibodies used were rabbit polyclonal anti-OXT (primary; 1:2000; Phoenix Pharmaceuticals #H-051-01) and goat anti-rabbit plus 555 (secondary; 1:1000; ThermoFisher #A32732).

### Light-sheet microscopy

Imaging was performed with resources available in the Zuckerman Institute’s Cellular Imaging Platform. Cleared and stained brains were imaged on a light-sheet microscope (Ultramicroscope II, Miltenyi Biotech) equipped with a sCMOS camera (Andor Neo) and a 2×/0.5 NA objective lens (MVPLAPO 2×). Brains were kept fully intact and imaged one hemisphere at a time. Samples were oriented sagittally such that the dorsal surface faced the right light-sheet, and only the right light-sheet, at 90% sheet width, was used for imaging. Tiles were set to overlap by 10%. Two acquisitions were completed per session: one for autofluorescence (488 nanometers channel) and one for c-Fos and OXT staining (647 nanometers and 555 nanometers, respectively and simultaneously). For autofluorescence, the following parameters were used: 0.63x zoom (1.26x effective magnification); no dynamic focus; 10 micrometer Z step-size; 50% laser power; 30 millisecond exposure time. For c-Fos and OXT, the following parameters were used: 0.8x zoom (1.6x effective magnification); 20 dynamic focus acquisitions per plane; three micrometer Z step-size; 90% laser power; 50 millisecond exposure time. All images were saved as 3D tiffs, and tiles were stitched together with the software Stitchy (Translucence Biosystems).

#### c-Fos+ cell counting

All c-Fos+ cell detection was performed with the open-source software ClearMap 2, available at https://github.com/ClearAnatomics/ClearMap (Renier et al., 2016). The settings used for cell detection were as follows: the background was removed by subtraction of the morphological opened image with a disk shape structure element with main axis of seven pixels in diameter. Cells were detected from peaks and subsequent watershedding, removing background pixels below an intensity cutoff of 700 and selecting cells with sizes between 20 and 500 voxels. These settings corresponded to ClearMap 2 cell detection parameters used in other studies, to facilitate future comparative analyses (Levy et al., 2019; Renier et al., 2016; Topilko et al., 2022). Samples were registered using the average autofluorescence STPR brain registered to the Allen Brain Institute 25 μm map, and its companion annotation map (http://alleninstitute.org/).

### c-Fos expression analysis

We simplified the Allen Brain Atlas down from 1,204 parcellations to 215 regions. This subset of 215 regions still tiled the whole brain, but truncated the granularity to exclude layer-specificity and combine parcellations that were deemed too small for our purposes. Given that the Allen Brain Atlas is symmetrical, c-Fos+ cell counts were summed across bilateral regions for all analyses except those that explicitly tested lateralization.

After using MATLAB (version 2022a) to collate how many c-Fos+ cells were detected per brain region of the 215 region subset, we used R (version 4.4.3) to model the data with generalized linear mixed models (GLMM). We fit a full GLMM for each brain region using the R package glmmTMB (Brooks et al., 2017), with a negative binomial link function and the formula:

RegioncFoscounts∼Stimulus∗Group+(1∣Batch)+ln(TotalsamplecFoscounts)

We fit a reduced GLMM for each brain region with this formula:

RegioncFoscounts∼(1∣Batch)+ln(TotalsamplecFoscounts)

The fixed-effect categorical variables included *Stimulus* (pup calls or baseline) and *Group* (naïve virgin, experienced virgin, or mother), with the asterisk representing both main effects and their interaction. *Batch* (1, 2, 3, or 4) was a random effect for each technical batch (each set of 12 samples that underwent tissue clearing, immunolabelling and light-sheet imaging together). *In(Total sample cFos counts)* was an offset term for the total number of c-Fos+ cells detected per sample. For each brain region, we fit the full and reduced model, then compared the fit of the 2 models using a likelihood-ratio chi-squared test and adjusted the resulting p-values using the Benjamini-Hochberg method to permit a 10% false discovery rate (FDR) across all brain regions.

For analyses aside from the GLMM, the number of c-Fos+ counts detected per brain region was divided by the total number of c-Fos+ cells detected within the whole sample. This within-sample normalization was done as an attempt to account for potential batch effects in antibody penetration and imaging. To assess which brain regions had a significant batch effect, even after this normalization, we conducted a 1-way ANOVA with Batch being the sole factor.

Following normalization by total sample count, each region’s c-Fos expression was then within-group baseline-standardized. This was achieved with the formula:

$$cFos^{PC}=\frac{x_{PC}-\mu_{B}}{\sigma_{B}}$$

Per brain region in a pup call-exposed sample, *x_PC_* is the observed c-Fos expression; *μ*_B_ is the average c-Fos expression in within-group baseline samples; *σ*_B_ is the standard deviation of c-Fos expression in within-group baseline samples.

### c-Fos expression correlation matrices

We used MATLAB (version 2022a) to compute, visualize, and compare Pearson correlation coefficients between pairs of brain regions, correlated over samples within the same condition, based on either (1) normalized c-Fos+ cell counts or (2) *cFos^PC^* values. To compare average interregional correlation across conditions, we transformed all interregional Pearson correlation coefficients using a Fisher r-to-z transformation, then conducted either one-way or two-way ANOVAs. To compare similarity between two correlation matrices, we conducted a Mantel test by vectorizing the upper triangle of each correlation matrix, excluding the diagonal, and calculating the Spearman’s correlation coefficient between the vectors.

### c-Fos expression correlation networks

Following the production of brain-wide *cFos^PC^* correlation matrices, we extracted the upper triangle of each matrix, excluding the diagonal, and applied a threshold to all pairwise correlations by their statistical significance, using a FDR of 0.05 and the Benjamini, Krieger, and Yekutieli two-stage step-up method (Benjamini et al., 2006) to correct for multiple comparisons across all pairwise correlations. The pairwise correlations depicted as weighted edges in network graphs all passed this thresholding, and these thresholded networks were then used for network analyses. Treating the resulting networks as unweighted graphs at first, network density was calculated using the formula:

$$Network\;density=\frac{\#\;existing\;edges}{\#\;total\;possible\;edges}$$

Still treating the networks as unweighted graphs, node degree was calculated per node, defined as the number of edges that node participates in. The average node degree per network was calculated across all 215 nodes. Finally, treating the networks as weighted graphs, we calculated average correlation magnitude by averaging the absolute values of all edges per network. To extract hub regions, each network’s nodes were sorted in descending degree order and the top 10% most-connected nodes out of the 215 total nodes were identified as hubs.

To compare network density, average node degree, and average correlation magnitude between the groups’ pup call response networks, we conducted three permutation tests (n = 1,000 permutations) by shuffling *cFos^PC^* data between (1) naïve virgins and experienced virgins, (2) naïve virgins and mothers, and (3) experienced virgins and mothers. For each permutation, we computed the difference between the two network’s densities, average node degrees, and average correlation magnitudes, and compared the observed differences to their respective null difference distributions to extract one-sided p-values.

### OXT projection mapping

OXT projections were characterized using a custom, consolidated deep learning-based analysis pipeline that automatically preprocesses, segments, and quantifies labeled projections across the whole brain using multiple MATLAB (version R2024b) and Python (version 3.14) scripts. This pipeline was based on TrailMap, developed to segment axonal projections from whole-brain light-sheet images, and DeepTraCE, which aligns TrailMap output to the Allen Brain Atlas and quantifies projection density (Friedmann et al., 2020; Gongwer et al., 2023). We used our pipeline to analyze the left hemisphere of each sample that was imaged left hemisphere first, to avoid any datasets with potential photobleaching. In brief, following brightness normalization and image chunking, we performed OXT projection segmentation with our custom deep learning-based segmentation model. The model was trained with a 3D U-Net architecture (Çiçek et al., 2016) in the open-source PyTorch deep learning framework with brightness-normalized image chunks. The loss function was an equally weighted combination of binary cross-entropy and squared Dice losses (Milletari et al., 2016). To overcome the need for a large amount of initial training data, we used the output of the TrailMap model on their publicly available serotoninergic data to train the foundation of our model. We then refined the model using our OXT training data, acquired by manually tracing entire chunks using the SNT neuroanatomy plugin on ImageJ (version 1.54p) (Arshadi et al., 2021). Following segmentation, image chunks were re-adjoined, and segmented images were binarized using probability threshold of 11 (out of 255), which we found maintained axonal integrity. The binary mask was then skeletonized, connected components were identified and thresholded by a voxel length of 10, and voxels that survived thresholding were deemed to be OXT-labeled. To identify where in the brain these OXT-labeled voxels were located, alignment to the Allen Brain Atlas was achieved via Elastix (Klein et al., 2010), using the same transformation parameters derived from running samples through the ClearMap 2 pipeline (Renier et al., 2016). After acquiring the spatial coordinates and granular region membership of OXT-labeled voxels, we assigned each OXT-labeled voxel to one region in our subset of 215 brain regions.

### OXT expression analysis

After using MATLAB (version 2022a) to collate how many OXT+ voxels were detected per brain region of the 215 region subset, we used R (version 4.4.3) to model the data with generalized linear mixed models (GLMM). We fit a full GLMM for each brain region using the R package glmmTMB (Brooks et al., 2017), with a negative binomial link function and the formula:

RegionOXTcounts∼Group+(1∣Batch)+ln(TotalsampleOXTcounts)

We fit a reduced GLMM for each brain region with this formula:

RegionOXTcounts∼(1∣Batch)+ln(TotalsampleOXTcounts)

The fixed-effect categorical variable included was *Group* (naïve virgin, experienced virgin, or mother). *Batch* (1, 2, 3, or 4) was a random effect for each technical batch (each set of 12 samples that underwent tissue clearing, immunolabelling and light-sheet imaging together). *In(Total sample OXT counts)* was an offset term for the total number of OXT+ voxels detected per sample. For each brain region, we fit the full and reduced model, then compared the fit of the 2 models using a likelihood-ratio chi-squared test and adjusted the resulting p-values using the Benjamini-Hochberg method to permit a 10% false discovery rate (FDR) across all brain regions.

For analyses aside from the GLMM, for each brain region, the number of OXT-labeled voxels was divided by the total number of OXT-labeled voxels in the full hemisphere, allowing us to capture the relative proportion of OXT projections present in this region compared to the whole hemisphere. We then calculated relative projection density per region by dividing that number by region volume in cubic millimeters. To define the OXT projection-dense (or sparse) circuit, the relative OXT projection density per region was averaged across all animals, and top (or bottom) 10% most (or least) dense regions were identified.

### Statistical analysis

Results were plotted and tested for statistical significance using MATLAB (versions 2022a and 2024b), R (version 4.4.3), and GraphPad Prism (version 10). Statistical tests are described in earlier Methods sections and figure legends.

## Extended Data

## Supplementary Material

Supplement 1

Supplement 2

Supplement 3

## Figures and Tables

**Table 1:** Allen Brain Atlas regions and abbreviations. For each of the 215 brain regions conserved from Allen Brain Atlas, this table lists their abbreviation, full name, and broader brain area membership.

#	Abbrev.	Name	Broader area membership
1	FRP	Frontal pole, cerebral cortex	Isocortex
2	MOp	Primary motor area	Isocortex
3	MOs	Secondary motor area	Isocortex
4	SSp	Primary somatosensory area	Isocortex
5	SSs	Supplemental somatosensory area	Isocortex
6	ILA	Infralimbic area	Isocortex
7	GU	Gustatory areas	Isocortex
8	VISC	Visceral area	Isocortex
9	AUDd	Dorsal auditory area	Isocortex
10	AUDp	Primary auditory area	Isocortex
11	AUDpo	Posterior auditory area	Isocortex
12	AUDv	Ventral auditory area	Isocortex
13	VISal	Anterolateral visual area	Isocortex
14	VISam	Anteromedial visual area	Isocortex
15	VISl	Lateral visual area	Isocortex
16	VISp	Primary visual area	Isocortex
17	VISpl	Posterolateral visual area	Isocortex
18	VISpm	Posteromedial visual area	Isocortex
19	ACAd	Anterior cingulate area, dorsal part	Isocortex
20	ACAv	Anterior cingulate area, ventral part	Isocortex
21	PL	Prelimbic area	Isocortex
22	ORBl	Orbital area, lateral part	Isocortex
23	ORBm	Orbital area, medial part	Isocortex
24	ORBvl	Orbital area, ventrolateral part	Isocortex
25	AId	Agranular insular area, dorsal part	Isocortex
26	AIp	Agranular insular area, posterior part	Isocortex
27	AIv	Agranular insular area, ventral part	Isocortex
28	RSPagl	Retrosplenial area, lateral agranular part	Isocortex
29	RSPd	Retrosplenial area, dorsal part	Isocortex
30	RSPv	Retrosplenial area, ventral part	Isocortex
31	TEa	Temporal association areas	Isocortex
32	PERI	Perirhinal area	Isocortex
33	ECT	Ectorhinal area	Isocortex
34	MOB	Main olfactory bulb	Olfactory areas
35	AOB	Accessory olfactory bulb	Olfactory areas
36	AON	Anterior olfactory nucleus	Olfactory areas
37	TT	Taenia tecta	Olfactory areas
38	DP	Dorsal peduncular area	Olfactory areas
39	PIR	Piriform area	Olfactory areas
40	NLOT	Nucleus of the lateral olfactory tract	Olfactory areas
41	COA	Cortical amygdalar area	Olfactory areas
42	PAA	Piriform-amygdalar area	Olfactory areas
43	TR	Postpiriform transition area	Olfactory areas
44	CA1	Field CA1	Hippocampal formation
45	CA2	Field CA2	Hippocampal formation
46	CA3	Field CA3	Hippocampal formation
47	DG	Dentate gyrus	Hippocampal formation
48	FC	Fasciola cinerea	Hippocampal formation
49	IG	Induseum griseum	Hippocampal formation
50	ENT	Entorhinal area	Hippocampal formation
51	PAR	Parasubiculum	Hippocampal formation
52	POST	Postsubiculum	Hippocampal formation
53	PRE	Presubiculum	Hippocampal formation
54	SUB	Subiculum	Hippocampal formation
55	CLA	Claustrum	Cortical subplate
56	EP	Endopiriform nucleus	Cortical subplate
57	LA	Lateral amygdalar nucleus	Cortical subplate
58	BLA	Basolateral amygdalar nucleus	Cortical subplate
59	BMA	Basomedial amygdalar nucleus	Cortical subplate
60	PA	Posterior amygdalar nucleus	Cortical subplate
61	CP	Caudoputamen	Striatum
62	ACB	Nucleus accumbens	Striatum
63	FS	Fundus of striatum	Striatum
64	OT	Olfactory tubercle	Striatum
65	LS	Lateral septal nucleus	Striatum
66	SF	Septofimbrial nucleus	Striatum
67	SH	Septohippocampal nucleus	Striatum
68	AAA	Anterior amygdalar area	Striatum
69	BA	Bed nucleus of the accessory olfactory tract	Striatum
70	CEA	Central amygdalar nucleus	Striatum
71	IA	Intercalated amygdalar nucleus	Striatum
72	MEA	Medial amygdalar nucleus	Striatum
73	GPe	Globus pallidus, external segment	Pallidum
74	GPi	Globus pallidus, internal segment	Pallidum
75	SI	Substantia innominata	Pallidum
76	MA	Magnocellular nucleus	Pallidum
77	MSC	Medial septal complex	Pallidum
78	TRS	Triangular nucleus of septum	Pallidum
79	BST	Bed nuclei of the stria terminalis	Pallidum
80	BAC	Bed nucleus of the anterior commissure	Pallidum
81	VERM	Vermal regions	Cerebellum
82	HEM	Hemispheric regions	Cerebellum
83	FN	Fastigial nucleus	Cerebellum
84	IP	Interposed nucleus	Cerebellum
85	DN	Dentate nucleus	Cerebellum
86	VENT	Ventral group of the dorsal thalamus	Thalamus
87	SPF	Subparafascicular nucleus	Thalamus
88	SPA	Subparafascicular area	Thalamus
89	PP	Peripeduncular nucleus	Thalamus
90	GENd	Geniculate group, dorsal thalamus	Thalamus
91	LAT	Lateral group of the dorsal thalamus	Thalamus
92	ATN	Anterior group of the dorsal thalamus	Thalamus
93	MED	Medial group of the dorsal thalamus	Thalamus
94	MTN	Midline group of the dorsal thalamus	Thalamus
95	ILM	Intralaminar nuclei of the dorsal thalamus	Thalamus
96	RT	Reticular nucleus of the thalamus	Thalamus
97	GENv	Geniculate group, ventral thalamus	Thalamus
98	EPI	Epithalamus	Thalamus
99	SO	Supraoptic nucleus	Hypothalamus
100	ASO	Accessory supraoptic group	Hypothalamus
101	PVH	Paraventricular hypothalamic nucleus	Hypothalamus
102	PVa	Periventricular hypothalamic nucleus, anterior part	Hypothalamus
103	PVi	Periventricular hypothalamic nucleus, intermediate part	Hypothalamus
104	ARH	Arcuate hypothalamic nucleus	Hypothalamus
105	ADP	Anterodorsal preoptic nucleus	Hypothalamus
106	AVP	Anteroventral preoptic nucleus	Hypothalamus
107	AVPV	Anteroventral periventricular nucleus	Hypothalamus
108	DMH	Dorsomedial nucleus of the hypothalamus	Hypothalamus
109	MEPO	Median preoptic nucleus	Hypothalamus
110	MPO	Medial preoptic area	Hypothalamus
111	OV	Vascular organ of the lamina terminalis	Hypothalamus
112	PD	Posterodorsal preoptic nucleus	Hypothalamus
113	PS	Parastrial nucleus	Hypothalamus
114	PVp	Periventricular hypothalamic nucleus, posterior part	Hypothalamus
115	PVpo	Periventricular hypothalamic nucleus, preoptic part	Hypothalamus
116	SBPV	Subparaventricular zone	Hypothalamus
117	SCH	Suprachiasmatic nucleus	Hypothalamus
118	SFO	Subfornical organ	Hypothalamus
119	VLPO	Ventrolateral preoptic nucleus	Hypothalamus
120	AHN	Anterior hypothalamic nucleus	Hypothalamus
121	MBO	Mammillary body	Hypothalamus
122	MPN	Medial preoptic nucleus	Hypothalamus
123	PMd	Dorsal premammillary nucleus	Hypothalamus
124	PMv	Ventral premammillary nucleus	Hypothalamus
125	PVHd	Paraventricular hypothalamic nucleus, descending division	Hypothalamus
126	VMH	Ventromedial hypothalamic nucleus	Hypothalamus
127	PH	Posterior hypothalamic nucleus	Hypothalamus
128	LHA	Lateral hypothalamic area	Hypothalamus
129	LPO	Lateral preoptic area	Hypothalamus
130	PST	Preparasubthalamic nucleus	Hypothalamus
131	PSTN	Parasubthalamic nucleus	Hypothalamus
132	RCH	Retrochiasmatic area	Hypothalamus
133	STN	Subthalamic nucleus	Hypothalamus
134	TU	Tuberal nucleus	Hypothalamus
135	ZI	Zona incerta	Hypothalamus
136	SCs	Superior colliculus, sensory related	Midbrain
137	IC	Inferior colliculus	Midbrain
138	NB	Nucleus of the brachium of the inferior colliculus	Midbrain
139	SAG	Nucleus sagulum	Midbrain
140	PBG	Parabigeminal nucleus	Midbrain
141	MEV	Midbrain trigeminal nucleus	Midbrain
142	SNr	Substantia nigra, reticular part	Midbrain
143	VTA	Ventral tegmental area	Midbrain
144	RR	Midbrain reticular nucleus, retrorubral area	Midbrain
145	MRN	Midbrain reticular nucleus	Midbrain
146	SCm	Superior colliculus, motor related	Midbrain
147	PAG	Periaqueductal gray	Midbrain
148	PRT	Pretectal region	Midbrain
149	CUN	Cuneiform nucleus	Midbrain
150	RN	Red nucleus	Midbrain
151	III	Oculomotor nucleus	Midbrain
152	EW	Edinger-Westphal nucleus	Midbrain
153	IV	Trochlear nucleus	Midbrain
154	VTN	Ventral tegmental nucleus	Midbrain
155	AT	Anterior tegmental nucleus	Midbrain
156	LT	Lateral terminal nucleus of the accessory optic tract	Midbrain
157	SNc	Substantia nigra, compact part	Midbrain
158	PPN	Pedunculopontine nucleus	Midbrain
159	RAmb	Midbrain raphe nuclei	Midbrain
160	NLL	Nucleus of the lateral lemniscus	Pons
161	PSV	Principal sensory nucleus of the trigeminal	Pons
162	PB	Parabrachial nucleus	Pons
163	SOC	Superior olivary complex	Pons
164	B	Barrington's nucleus	Pons
165	DTN	Dorsal tegmental nucleus	Pons
166	PCG	Pontine central gray	Pons
167	PG	Pontine gray	Pons
168	PRNc	Pontine reticular nucleus, caudal part	Pons
169	SG	Supragenual nucleus	Pons
170	SUT	Supratrigeminal nucleus	Pons
171	TRN	Tegmental reticular nucleus	Pons
172	V	Motor nucleus of trigeminal	Pons
173	CS	Superior central nucleus raphe	Pons
174	LC	Locus ceruleus	Pons
175	LDT	Laterodorsal tegmental nucleus	Pons
176	NI	Nucleus incertus	Pons
177	PRNr	Pontine reticular nucleus	Pons
178	RPO	Nucleus raph√© pontis	Pons
179	SLC	Subceruleus nucleus	Pons
180	SLD	Sublaterodorsal nucleus	Pons
181	AP	Area postrema	Medulla
182	CN	Cochlear nuclei	Medulla
183	DCN	Dorsal column nuclei	Medulla
184	ECU	External cuneate nucleus	Medulla
185	NTB	Nucleus of the trapezoid body	Medulla
186	NTS	Nucleus of the solitary tract	Medulla
187	SPVC	Spinal nucleus of the trigeminal, caudal part	Medulla
188	SPVI	Spinal nucleus of the trigeminal, interpolar part	Medulla
189	SPVO	Spinal nucleus of the trigeminal, oral part	Medulla
190	VI	Abducens nucleus	Medulla
191	VII	Facial motor nucleus	Medulla
192	ACVII	Accessory facial motor nucleus	Medulla
193	AMB	Nucleus ambiguus	Medulla
194	DMX	Dorsal motor nucleus of the vagus nerve	Medulla
195	GRN	Gigantocellular reticular nucleus	Medulla
196	ICB	Infracerebellar nucleus	Medulla
197	IO	Inferior olivary complex	Medulla
198	IRN	Intermediate reticular nucleus	Medulla
199	ISN	Inferior salivatory nucleus	Medulla
200	LIN	Linear nucleus of the medulla	Medulla
201	LRN	Lateral reticular nucleus	Medulla
202	MARN	Magnocellular reticular nucleus	Medulla
203	MDRN	Medullary reticular nucleus	Medulla
204	PARN	Parvicellular reticular nucleus	Medulla
205	PAS	Parasolitary nucleus	Medulla
206	PGRN	Paragigantocellular reticular nucleus	Medulla
207	PHY	Perihypoglossal nuclei	Medulla
208	PPY	Parapyramidal nucleus	Medulla
209	VNC	Vestibular nuclei	Medulla
210	x	Nucleus x	Medulla
211	XII	Hypoglossal nucleus	Medulla
212	y	Nucleus y	Medulla
213	RM	Nucleus raphe magnus	Medulla
214	RPA	Nucleus raphe pallidus	Medulla
215	RO	Nucleus raphe obscurus	Medulla

**Table 2:** GLMM results. Results from fitting a full and reduced GLMM (see Methods) per brain region, and comparing the fit of the 2 models. Significant p-value would indicate that full GLMM explains the c-Fos+ counts in that brain region better than the reduced GLMM. Middle column indicates p-value of model comparison (green shading highlights regions with p < 0.05), rightmost column indicates adjusted p-value of model comparison (red shading highlights regions with FDR < 0.10).

Region	p-value from model comparison	Adjusted p-value (FDR < 0.10)
FRP	0.86287857	0.92568707
MOp	0.07578609	0.68099235
MOs	0.77321023	0.8883698
SSp	0.37586341	0.79443153
SSs	0.70608113	0.8883698
ILA	0.47035576	0.88106721
GU	0.02492231	0.68099235
VISC	0.54794771	0.8883698
AUDd	0.28344322	0.73477471
AUDp	0.18566147	0.68099235
AUDpo	0.01198419	0.68099235
AUDv	0.8677685	0.92568707
VISal	0.13984382	0.68099235
VISam	0.62675302	0.8883698
VISl	8.81549290463625e-06	0.00189533
VISp	0.57569667	0.8883698
VISpl	0.00012337	0.01326226
VISpm	0.77733329	0.8883698
ACAd	0.32909511	0.76754788
ACAv	0.51974454	0.88686569
PL	0.65680196	0.8883698
ORBl	0.64396994	0.8883698
ORBm	0.19784725	0.68099235
ORBvl	0.24070838	0.72049635
AId	0.5657859	0.8883698
AIp	0.09555557	0.68099235
AIv	0.87402082	0.92568707
RSPagl	0.74845858	0.8883698
RSPd	0.5869099	0.8883698
RSPv	0.12006543	0.68099235
TEa	0.05687867	0.68099235
PERI	0.23879685	0.72049635
ECT	0.35773909	0.79292686
MOB	0.8373986	0.92425134
AOB	0.54683733	0.8883698
AON	0.74570139	0.8883698
TT	0.72137967	0.8883698
DP	0.57927024	0.8883698
PIR	0.2786311	0.73477471
NLOT	0.75866684	0.8883698
COA	0.1459944	0.68099235
PAA	0.89317323	0.927692
TR	0.66819247	0.8883698
CA1	0.7509442	0.8883698
CA2	0.92710861	0.94918263
CA3	0.54114115	0.8883698
DG	0.75333551	0.8883698
FC	0.03812106	0.68099235
IG	0.31225872	0.75031315
ENT	0.18954748	0.68099235
PAR	0.78093903	0.8883698
POST	0.18383133	0.68099235
PRE	0.36906772	0.7934956
SUB	0.19659414	0.68099235
CLA	0.48196758	0.88106721
EP	0.87945236	0.92569342
LA	0.44771134	0.88106721
BLA	0.1899192	0.68099235
BMA	0.24965274	0.72049635
PA	0.09762295	0.68099235
CP	0.57714496	0.8883698
ACB	0.94685232	0.95574295
FS	0.61834115	0.8883698
OT	0.59648883	0.8883698
LS	0.70839075	0.8883698
SF	0.944892	0.95574295
SH	0.71849869	0.8883698
AAA	0.67215393	0.8883698
BA	0.99317296	0.99317296
CEA	0.6491498	0.8883698
IA	0.72629283	0.8883698
MEA	0.69154274	0.8883698
GPe	0.15105994	0.68099235
GPi	0.48636662	0.88106721
SI	0.3768931	0.79443153
MA	0.49201751	0.88106721
MSC	0.15335546	0.68099235
TRS	0.61040589	0.8883698
BST	0.77869919	0.8883698
BAC	0.07513395	0.68099235
VERM	0.66988722	0.8883698
HEM	0.38479574	0.79925121
FN	0.8707758	0.92568707
IP	0.34144798	0.77275069
DN	0.07214301	0.68099235
VENT	0.0577405	0.68099235
SPF	0.25803823	0.72049635
SPA	0.56247209	0.8883698
PP	0.24727162	0.72049635
GENd	0.61882552	0.8883698
LAT	0.91693354	0.94779188
ATN	0.22862593	0.72049635
MED	0.18615219	0.68099235
MTN	0.49773969	0.88106721
ILM	0.13715774	0.68099235
RT	0.11118662	0.68099235
GENv	0.73897951	0.8883698
EPI	0.57211001	0.8883698
SO	0.69577456	0.8883698
ASO	0.5958894	0.8883698
PVH	0.34695293	0.77703001
PVa	0.75175345	0.8883698
PVi	0.80608841	0.8982942
ARH	0.51554683	0.88674055
ADP	0.83956841	0.92425134
AVP	0.29739471	0.73944458
AVPV	0.17861813	0.68099235
DMH	0.2577635	0.72049635
MEPO	0.08228319	0.68099235
MPO	0.58432967	0.8883698
OV	0.13424841	0.68099235
PD	0.72868802	0.8883698
PS	0.33200908	0.76754788
PVp	0.72895533	0.8883698
PVpo	0.25648885	0.72049635
SBPV	0.09922299	0.68099235
SCH	0.01831757	0.68099235
SFO	0.4179075	0.84764257
VLPO	0.38661454	0.79925121
AHN	0.15129479	0.68099235
MBO	0.17404181	0.68099235
MPN	0.21502807	0.72049635
PMd	0.29921711	0.73944458
PMv	0.49183504	0.88106721
PVHd	0.4850519	0.88106721
VMH	0.13670046	0.68099235
PH	0.54422189	0.8883698
LHA	0.50258828	0.88106721
LPO	0.36192686	0.7934956
PST	0.28875645	0.73907902
PSTN	0.44614136	0.88106721
RCH	0.19954659	0.68099235
STN	0.77705393	0.8883698
TU	0.31215862	0.75031315
ZI	0.18127427	0.68099235
SCs	0.22471443	0.72049635
IC	0.62416213	0.8883698
NB	0.76703956	0.8883698
SAG	0.47284921	0.88106721
PBG	0.32191668	0.76057238
MEV	0.80637573	0.8982942
SNr	0.55306018	0.8883698
VTA	0.04827669	0.68099235
RR	0.46660767	0.88106721
MRN	0.16134574	0.68099235
SCm	0.29301735	0.73944458
PAG	0.36685448	0.7934956
PRT	0.78587442	0.88927895
CUN	0.720967	0.8883698
RN	0.01900347	0.68099235
III	0.14009508	0.68099235
EW	0.31408457	0.75031315
IV	0.12280988	0.68099235
VTN	0.64096965	0.8883698
AT	0.56438108	0.8883698
LT	0.49201572	0.88106721
SNc	0.16412472	0.68099235
PPN	0.74040045	0.8883698
RAmb	0.10759762	0.68099235
NLL	0.16371381	0.68099235
PSV	0.13534368	0.68099235
PB	0.92443401	0.94918263
SOC	0.53599409	0.8883698
B	0.50405241	0.88106721
DTN	0.51039665	0.88496194
PCG	0.6068473	0.8883698
PG	0.26784257	0.73477471
PRNc	0.22967185	0.72049635
SG	0.34093633	0.77275069
SUT	0.28365721	0.73477471
TRN	0.05716134	0.68099235
V	0.72787302	0.8883698
CS	0.88694346	0.92569342
LC	0.75745057	0.8883698
LDT	0.72218454	0.8883698
NI	0.88392194	0.92569342
PRNr	0.16267473	0.68099235
RPO	0.9618055	0.96629992
SLC	0.05480754	0.68099235
SLD	0.07313899	0.68099235
AP	0.93567527	0.95341319
CN	0.02749249	0.68099235
DCN	0.71573777	0.8883698
ECU	0.67451466	0.8883698
NTB	0.49992408	0.88106721
NTS	0.1491332	0.68099235
SPVC	0.14583462	0.68099235
SPVI	0.18030031	0.68099235
SPVO	0.08324099	0.68099235
VI	0.60722706	0.8883698
VII	0.84257332	0.92425134
ACVII	0.0843698	0.68099235
AMB	0.1217536	0.68099235
DMX	0.86944024	0.92568707
GRN	0.19865812	0.68099235
ICB	0.09591202	0.68099235
IO	0.74087088	0.8883698
IRN	0.24369931	0.72049635
ISN	0.28309632	0.73477471
LIN	0.03861695	0.68099235
LRN	0.80472006	0.8982942
MARN	0.28047629	0.73477471
MDRN	0.85927474	0.92568707
PARN	0.43728046	0.87864765
PAS	0.13937487	0.68099235
PGRN	0.40355214	0.82632104
PHY	0.1619317	0.68099235
PPY	0.49863843	0.88106721
VNC	0.22522705	0.72049635
x	0.59156201	0.8883698
XII	0.85745385	0.92568707
y	0.06244422	0.68099235
RM	0.25358023	0.72049635
RPA	0.12041529	0.68099235
RO	0.72485467	0.8883698

**Table 3:** 2-way ANOVA results on brain-wide average interregional correlation. Tukey’s post-hoc tests on main comparisons of interest from the bar graph in Figure 2.

Group 1	Group 2	p-value
Naïve virgins + baseline	Naïve virgins + pup calls	< 0.0001 (****)
Experienced virgins + baseline	Experienced virgins + pup calls	0.0001796 (***)
Mothers + baseline	Mothers + pup calls	0.0004070 (***)
Naïve virgins + baseline	Experienced virgins + baseline	< 0.0001 (****)
Naïve virgins + baseline	Mothers + baseline	< 0.0001 (****)
Experienced virgins + baseline	Mothers + baseline	0.347 (ns)
Naïve virgins + pup calls	Experienced virgins + pup calls	< 0.0001 (****)
Naïve virgins + pup calls	Mothers + pup calls	< 0.0001 (****)
Experienced virgins + pup calls	Mothers + pup calls	0.4611 (ns)

**Table 4:** Node degree per each group’s pup call response network. For each of the 215 brain regions conserved from Allen Brain Atlas, this table lists their abbreviation (see Table 1 for abbreviations) and node degree in our pup call response networks per group, in descending degree order.

Naïve virgins		Experienced virgins		Mothers	
*Region*	*Node* *degree*	*Region*	*Node* *degree*	*Region*	*Node* *degree*
GPi	29	BA	12	AHN	3
VENT	28	LHA	11	PERI	2
ZI	27	AAA	10	BLA	2
AAA	22	FS	8	FS	2
LPO	22	IA	8	BA	2
ILM	21	MA	8	CEA	2
SO	21	LPO	7	IA	2
LHA	21	NLOT	6	MEA	2
ATN	20	MEA	6	VENT	2
SFO	20	GPe	6	SO	2
IA	19	BLA	5	PVp	2
MTN	18	CEA	5	LHA	2
PVH	18	BMA	4	DMX	2
GENv	16	MTN	4	PPY	2
TU	16	SO	4	MOp	1
LA	15	RCH	4	MOs	1
TRS	15	TU	4	VISC	1
SPF	15	GPi	3	AIp	1
RCH	15	ATN	3	NLOT	1
CEA	14	ILM	3	COA	1
SPA	14	RT	3	PAA	1
RT	14	MEV	3	BMA	1
AHN	14	AIp	2	AAA	1
CA3	13	PRE	2	ATN	1
BA	13	SF	2	MBO	1
BST	13	SPA	2	PMd	1
MED	13	ASO	2	PMv	1
MEA	12	AVP	2	PG	1
NLOT	11	MEPO	2	TRN	1
BLA	11	ZI	2	CN	1
VMH	11	SCs	2	DCN	1
FS	10	IC	2	PARN	1
ASO	9	B	2	PHY	1
CA2	8	PCG	2	FRP	0
BMA	8	MARN	2	SSp	0
MA	8	RM	2	SSs	0
MPN	8	RPA	2	ILA	0
TT	7	VISC	1	GU	0
PVi	7	VISl	1	AUDd	0
MPO	7	VISpl	1	AUDp	0
SBPV	6	COA	1	AUDpo	0
PH	6	DG	1	AUDv	0
AUDp	5	EP	1	VISal	0
AUDpo	5	LA	1	VISam	0
VISl	5	TRS	1	VISl	0
PL	5	FN	1	VISp	0
IG	5	VENT	1	VISpl	0
ORBl	4	SPF	1	VISpm	0
TEa	4	EPI	1	ACAd	0
ENT	4	PS	1	ACAv	0
PRE	4	PVpo	1	PL	0
SF	4	PMd	1	ORBl	0
PVa	4	PMv	1	ORBm	0
DMH	4	PSTN	1	ORBvl	0
PS	4	SCm	1	AId	0
VLPO	4	AT	1	AIv	0
PPY	4	PSV	1	RSPagl	0
AUDv	3	SOC	1	RSPd	0
VISal	3	DTN	1	RSPv	0
ORBm	3	LC	1	TEa	0
AId	3	IRN	1	ECT	0
DP	3	PARN	1	MOB	0
EP	3	FRP	0	AOB	0
LS	3	MOp	0	AON	0
LAT	3	MOs	0	TT	0
SCH	3	SSp	0	DP	0
B	3	SSs	0	PIR	0
SSp	2	ILA	0	TR	0
AUDd	2	GU	0	CA1	0
CLA	2	AUDd	0	CA2	0
PA	2	AUDp	0	CA3	0
HEM	2	AUDpo	0	DG	0
PSTN	2	AUDv	0	FC	0
STN	2	VISal	0	IG	0
PBG	2	VISam	0	ENT	0
LT	2	VISp	0	PAR	0
RAmb	2	VISpm	0	POST	0
LDT	2	ACAd	0	PRE	0
ECU	2	ACAv	0	SUB	0
NTS	2	PL	0	CLA	0
DMX	2	ORBl	0	EP	0
PAS	2	ORBm	0	LA	0
PHY	2	ORBvl	0	PA	0
XII	2	AId	0	CP	0
RM	2	AIv	0	ACB	0
SSs	1	RSPagl	0	OT	0
ILA	1	RSPd	0	LS	0
VISpl	1	RSPv	0	SF	0
ORBvl	1	TEa	0	SH	0
AIp	1	PERI	0	GPe	0
AOB	1	ECT	0	GPi	0
AON	1	MOB	0	SI	0
COA	1	AOB	0	MA	0
PAA	1	AON	0	MSC	0
DG	1	TT	0	TRS	0
FC	1	DP	0	BST	0
CP	1	PIR	0	BAC	0
ACB	1	PAA	0	VERM	0
GPe	1	TR	0	HEM	0
VERM	1	CA1	0	FN	0
IP	1	CA2	0	IP	0
ARH	1	CA3	0	DN	0
PVHd	1	FC	0	SPF	0
PAG	1	IG	0	SPA	0
VTN	1	ENT	0	PP	0
PSV	1	PAR	0	GENd	0
DTN	1	POST	0	LAT	0
PCG	1	SUB	0	MED	0
SG	1	CLA	0	MTN	0
LC	1	PA	0	ILM	0
RPO	1	CP	0	RT	0
VI	1	ACB	0	GENv	0
ACVII	1	OT	0	EPI	0
AMB	1	LS	0	ASO	0
ICB	1	SH	0	PVH	0
IO	1	SI	0	PVa	0
ISN	1	MSC	0	PVi	0
LRN	1	BST	0	ARH	0
y	1	BAC	0	ADP	0
FRP	0	VERM	0	AVP	0
MOp	0	HEM	0	AVPV	0
MOs	0	IP	0	DMH	0
GU	0	DN	0	MEPO	0
VISC	0	PP	0	MPO	0
VISam	0	GENd	0	OV	0
VISp	0	LAT	0	PD	0
VISpm	0	MED	0	PS	0
ACAd	0	GENv	0	PVpo	0
ACAv	0	PVH	0	SBPV	0
AIv	0	PVa	0	SCH	0
RSPagl	0	PVi	0	SFO	0
RSPd	0	ARH	0	VLPO	0
RSPv	0	ADP	0	MPN	0
PERI	0	AVPV	0	PVHd	0
ECT	0	DMH	0	VMH	0
MOB	0	MPO	0	PH	0
PIR	0	OV	0	LPO	0
TR	0	PD	0	PST	0
CA1	0	PVp	0	PSTN	0
PAR	0	SBPV	0	RCH	0
POST	0	SCH	0	STN	0
SUB	0	SFO	0	TU	0
OT	0	VLPO	0	ZI	0
SH	0	AHN	0	SCs	0
SI	0	MBO	0	IC	0
MSC	0	MPN	0	NB	0
BAC	0	PVHd	0	SAG	0
FN	0	VMH	0	PBG	0
DN	0	PH	0	MEV	0
PP	0	PST	0	SNr	0
GENd	0	STN	0	VTA	0
EPI	0	NB	0	RR	0
ADP	0	SAG	0	MRN	0
AVP	0	PBG	0	SCm	0
AVPV	0	SNr	0	PAG	0
MEPO	0	VTA	0	PRT	0
OV	0	RR	0	CUN	0
PD	0	MRN	0	RN	0
PVp	0	PAG	0	III	0
PVpo	0	PRT	0	EW	0
MBO	0	CUN	0	IV	0
PMd	0	RN	0	VTN	0
PMv	0	III	0	AT	0
PST	0	EW	0	LT	0
SCs	0	IV	0	SNc	0
IC	0	VTN	0	PPN	0
NB	0	LT	0	RAmb	0
SAG	0	SNc	0	NLL	0
MEV	0	PPN	0	PSV	0
SNr	0	RAmb	0	PB	0
VTA	0	NLL	0	SOC	0
RR	0	PB	0	B	0
MRN	0	PG	0	DTN	0
SCm	0	PRNc	0	PCG	0
PRT	0	SG	0	PRNc	0
CUN	0	SUT	0	SG	0
RN	0	TRN	0	SUT	0
III	0	V	0	V	0
EW	0	CS	0	CS	0
IV	0	LDT	0	LC	0
AT	0	NI	0	LDT	0
SNc	0	PRNr	0	NI	0
PPN	0	RPO	0	PRNr	0
NLL	0	SLC	0	RPO	0
PB	0	SLD	0	SLC	0
SOC	0	AP	0	SLD	0
PG	0	CN	0	AP	0
PRNc	0	DCN	0	ECU	0
SUT	0	ECU	0	NTB	0
TRN	0	NTB	0	NTS	0
V	0	NTS	0	SPVC	0
CS	0	SPVC	0	SPVI	0
NI	0	SPVI	0	SPVO	0
PRNr	0	SPVO	0	VI	0
SLC	0	VI	0	VII	0
SLD	0	VII	0	ACVII	0
AP	0	ACVII	0	AMB	0
CN	0	AMB	0	GRN	0
DCN	0	DMX	0	ICB	0
NTB	0	GRN	0	IO	0
SPVC	0	ICB	0	IRN	0
SPVI	0	IO	0	ISN	0
SPVO	0	ISN	0	LIN	0
VII	0	LIN	0	LRN	0
GRN	0	LRN	0	MARN	0
IRN	0	MDRN	0	MDRN	0
LIN	0	PAS	0	PAS	0
MARN	0	PGRN	0	PGRN	0
MDRN	0	PHY	0	VNC	0
PARN	0	PPY	0	x	0
PGRN	0	VNC	0	XII	0
VNC	0	x	0	y	0
x	0	XII	0	RM	0
RPA	0	y	0	RPA	0
RO	0	RO	0	RO	0

**Table 5:** Correlation magnitude per each group’s pup call response network. For each edge, this table lists the 2 regions’ abbreviation (see Table 1 for abbreviations) and their correlation magnitude in descending magnitude order.

Naïve virgins			Experienced virgins			Mothers		
*Region* *1*	*Region* *2*	∣*r*∣	*Region* *1*	*Region* *2*	∣*r*∣	*Region* *1*	*Region* *2*	∣*r*∣
FS	SPA	0.9952591	GPi	RT	0.99472199	MEA	LHA	0.99654035
GPi	ZI	0.99491456	AAA	BA	0.99432302	VENT	PPY	0.99612666
SPA	LPO	0.99460961	BMA	MEA	0.99238565	CEA	MEA	0.9949246
ATN	MTN	0.99439707	FS	IA	0.99061804	CN	PARN	0.99357551
GPi	VENT	0.99434908	IA	LHA	0.98997118	CEA	LHA	0.99318389
ECU	NTS	0.99387602	VENT	ZI	0.98990426	PERI	VENT	0.98699671
GENv	SFO	0.99276357	FS	LHA	0.9898262	FS	BA	0.98632598
ASO	PH	0.99209735	SCs	PSV	0.98949698	MOp	MOs	0.9852229
ECU	PHY	0.99202644	IA	MEA	0.98926609	PERI	PPY	0.98488693
VENT	ZI	0.99040328	PRE	RPA	0.98917445	NLOT	AAA	0.9847623
RCH	TU	0.99028434	AAA	LHA	0.98866741	IA	SO	0.98469854
LA	VMH	0.9894186	PVpo	IC	0.98781784	FS	AHN	0.98391291
CEA	LHA	0.98894142	PMd	PMv	0.98764963	COA	PAA	0.9829132
MED	RT	0.98748092	BA	CEA	0.98763123	IA	DMX	0.98174173
AAA	ILM	0.98715511	MEA	TU	0.98702168	PVp	PMd	0.98082613
BMA	TU	0.98669667	BA	LHA	0.98682915	SO	DMX	0.98051936
ILM	SFO	0.98612156	SO	ASO	0.98628052	VISC	AIp	0.97856717
VENT	LHA	0.98601948	CEA	RCH	0.98509411	BLA	BMA	0.97816897
MTN	ILM	0.98580413	MEV	SCm	0.98413823	PVp	PMv	0.97692784
TRS	ZI	0.98541654	ILM	RT	0.98340026	ATN	AHN	0.97688804
ATN	ILM	0.98523099	GPi	ILM	0.98188737	BLA	DCN	0.97642629
MED	ILM	0.98438092	CEA	ATN	0.98169254	MBO	PHY	0.9741497
NTS	PHY	0.98368391	PRE	MEV	0.98152868	PG	TRN	0.97364022
ILM	RT	0.98355037	DG	EPI	0.97916482	BA	AHN	0.97284977
AAA	LPO	0.98347653	SO	LPO	0.97905518			
LT	DMX	0.98346252	NLOT	LHA	0.97819672			
PVi	SBPV	0.98318204	BA	LPO	0.97802404			
IA	VENT	0.9830418	BMA	IA	0.97798901			
IA	ZI	0.98272906	MEV	RPA	0.97758972			
SPA	SO	0.98237542	FS	AAA	0.97671324			
MA	VLPO	0.98196213	CEA	LHA	0.97618839			
GPi	LHA	0.98192152	AAA	CEA	0.97555113			
MTN	RT	0.98183476	SPA	MTN	0.97545325			
PVi	VMH	0.98181379	NLOT	FS	0.97534644			
FS	LPO	0.98175226	AT	DTN	0.97519767			
SFO	ZI	0.98144224	BA	MA	0.97518303			
FS	SO	0.98109781	PS	LPO	0.97457085			
VISl	RM	0.98087608	COA	BLA	0.97426579			
GPi	SFO	0.98068408	BA	SO	0.97369113			
TRS	MED	0.98059598	NLOT	IA	0.97342729			
MEA	TU	0.97993239	GPi	MTN	0.97272578			
PVi	DMH	0.97975789	MEPO	SOC	0.97259141			
NLOT	IA	0.97962748	AAA	SO	0.9724462			
IA	MEA	0.97937728	B	PCG	0.97223253			
PA	ISN	0.9790929	FN	LC	0.97214086			
NLOT	MEA	0.97900433	MTN	RT	0.97183629			
BST	RCH	0.97872866	LA	BLA	0.97159017			
AAA	SO	0.97819026	BLA	BMA	0.97158871			
AAA	SPA	0.97807482	BA	ATN	0.97126504			
TRS	ILM	0.97791398	AAA	IA	0.97110547			
NLOT	SPF	0.97777278	BA	RCH	0.97105915			
VENT	ILM	0.97757837	AAA	RCH	0.97089587			
AHN	LHA	0.97749651	FS	BA	0.97077276			
ILM	ZI	0.97724338	AAA	LPO	0.97038993			
AAA	SFO	0.97630995	NLOT	AAA	0.97031217			
MEA	LHA	0.97630575	MEPO	IC	0.97002632			
TRS	RT	0.976241	MEA	LHA	0.96999558			
TRS	SFO	0.97556335	GPe	MA	0.96963311			
VENT	SFO	0.97553939	SPF	ZI	0.9696289			
PH	PPY	0.97553101	MA	LHA	0.96887988			
ATN	RT	0.9749498	LHA	RCH	0.96855914			
DMH	SBPV	0.97485407	SCs	RM	0.96829362			
IA	GPi	0.97434058	GPe	LHA	0.96808389			
PVH	AHN	0.97389966	BMA	TU	0.96742229			
LHA	ZI	0.97383621	BA	GPe	0.96707846			
MED	MTN	0.97374021	MARN	RM	0.96658407			
MEA	BST	0.97354425	NLOT	MA	0.96612243			
RT	ZI	0.97330109	VISC	AIp	0.96563265			
BMA	RCH	0.97327508	AAA	MA	0.96531968			
AAA	IA	0.97322876	FS	MA	0.96457409			
MA	SPA	0.9731978	MA	LPO	0.96420684			
HEM	PSTN	0.97273064	BLA	MEA	0.96337629			
SO	LPO	0.97260045	GPe	SPA	0.96273893			
CEA	AHN	0.97225469	GPe	ATN	0.9626597			
MA	LPO	0.97225467	SF	TRS	0.96263163			
AUDp	AUDpo	0.97217598	IA	TU	0.96257061			
BA	SO	0.97204873	FS	GPe	0.96243871			
MEA	VENT	0.97204273	BLA	TU	0.96142944			
VENT	RT	0.9719636	AIp	PSTN	0.96115449			
CEA	MEA	0.97191415	FS	MEA	0.96106396			
IA	ILM	0.97186286	BA	IA	0.96085709			
ILM	SO	0.97157189	IRN	PARN	0.95915673			
MEA	RCH	0.97148221	MTN	ILM	0.95896409			
ATN	SFO	0.97147958	LHA	LPO	0.95851458			
CA3	GPi	0.97088741	NLOT	BA	0.95846941			
ATN	MED	0.97077736	ASO	LPO	0.95807675			
DMX	XII	0.97055248	VISpl	MARN	0.95804401			
RT	SFO	0.97048505	SF	MA	0.95788101			
MTN	SFO	0.97043519	VISl	EP	−0.9577848			
GPi	ILM	0.97027041	AVP	B	−0.9595474			
CA2	RCH	0.97016889	AVP	PCG	−0.9636026			
AUDpo	VISal	0.96957952						
TEa	ENT	0.96949133						
ILA	PL	0.96941203						
MEA	ZI	0.96906687						
ILM	GENv	0.96906262						
GPi	TRS	0.96903616						
BST	TU	0.96875415						
MTN	GENv	0.96827329						
AAA	VENT	0.96823413						
MEA	GPi	0.96815153						
CEA	MPN	0.96804623						
GPi	RT	0.96782524						
ATN	GENv	0.96750114						
MPO	MPN	0.96743018						
IA	SPF	0.96719969						
LHA	RCH	0.96692519						
RT	GENv	0.96690115						
VERM	IP	0.96668883						
LT	XII	0.96662342						
CA3	STN	0.96660385						
SBPV	VMH	0.96607217						
TT	DP	0.96561784						
MED	ZI	0.96560196						
TU	ZI	0.96518505						
MPO	PS	0.96516151						
PS	MPN	0.96508606						
SO	ASO	0.96462351						
GPi	GENv	0.96459797						
MTN	SO	0.96456062						
AAA	MTN	0.96451871						
ORBvl	AON	0.96442254						
TRS	GENv	0.96439375						
FS	AAA	0.96430388						
ATN	SO	0.96401986						
SFO	LPO	0.96395729						
GENv	ZI	0.96351926						
AAA	ZI	0.96351911						
PVH	SFO	0.96348979						
PVHd	AMB	0.96253001						
MPO	LPO	0.96226878						
CA2	BLA	0.96224772						
BA	IA	0.96221187						
AHN	MPN	0.96207821						
IA	MED	0.96198174						
BLA	TU	0.96185937						
LA	SCH	0.96166177						
VMH	RCH	0.96161336						
TRS	VENT	0.96151825						
AAA	MED	0.96140966						
ASO	PPY	0.96136166						
SO	SFO	0.96117071						
ORBl	ORBm	0.96093567						
MEA	SPF	0.96082241						
ATN	PH	0.96052096						
B	LC	0.96038324						
PVa	PVi	0.96014675						
CEA	VENT	0.96003441						
MED	SFO	0.95995925						
LA	RCH	0.95979027						
CA3	VENT	0.95952875						
ILM	LPO	0.95927037						
AAA	ATN	0.95898671						
AId	DP	0.95876644						
IA	TRS	0.95855717						
SO	PVH	0.95845407						
CA2	TU	0.95844856						
BST	LHA	0.95840109						
BA	VENT	0.95828109						
VENT	SO	0.95820454						
BMA	MEA	0.95818082						
GPi	TU	0.95808833						
IA	LHA	0.95797625						
CEA	BST	0.95774687						
BMA	BST	0.9571536						
AUDv	TEa	0.95700799						
SPF	ZI	0.95691215						
PVH	LHA	0.95668296						
PVH	LPO	0.95649433						
ICB	PAS	0.95648235						
GPi	RCH	0.95621026						
CA3	BA	0.95617482						
FS	MA	0.95613224						
AUDd	VISal	0.95587856						
SPA	MPO	0.95585075						
GPi	SPF	0.95577861						
VMH	LHA	0.95553031						
LA	GPi	0.95530294						
MA	MPO	0.95504784						
MTN	PH	0.95501881						
AAA	GPi	0.95471232						
RCH	ZI	0.95463573						
SFO	LHA	0.95445523						
IA	RT	0.95414325						
NLOT	TU	0.95405248						
AAA	BA	0.9539877						
IA	SFO	0.95393571						
SSs	DG	0.95388305						
SSp	CA3	0.95387948						
VENT	GENv	0.95377795						
PAG	LDT	0.95375554						
VENT	PVH	0.9535483						
LAT	ATN	0.95350035						
LHA	TU	0.95342703						
NLOT	ZI	0.9529778						
VENT	MED	0.95288927						
AAA	RT	0.95268315						
LA	PVi	0.95255334						
AUDpo	VISl	0.95216683						
ATN	PPY	0.95213251						
CA2	BST	0.95210774						
DMH	VMH	0.95205786						
PL	RAmb	0.95201317						
AAA	TRS	0.95181913						
TRS	ATN	0.95177257						
MTN	PPY	0.95174492						
IA	TU	0.95172339						
BA	ILM	0.95160261						
AAA	GENv	0.95126826						
VENT	AHN	0.95125246						
TRS	MTN	0.95104375						
GENv	PVH	0.95103017						
BLA	BMA	0.95055784						
SBPV	AHN	0.95029013						
AHN	VMH	0.95010062						
CLA	GPe	0.94999635						
ILM	PVH	0.94999084						
CA2	BMA	0.94980978						
CEA	GPi	0.94978474						
FS	ASO	0.94977563						
CA3	ZI	0.94974937						
SG	VI	0.94942935						
SPA	PVH	0.94927656						
VENT	TU	0.94899082						
AAA	MA	0.94897738						
NLOT	BST	0.94895451						
MED	GENv	0.94877833						
ATN	ASO	0.94875195						
LA	LHA	0.94828904						
SPA	ILM	0.94811604						
CEA	RCH	0.94795125						
SPA	SFO	0.94772691						
GPi	AHN	0.94769869						
VENT	MTN	0.94769417						
GPi	MED	0.94768776						
VENT	SPF	0.94679307						
SPA	ASO	0.94665624						
AAA	SPF	0.94653553						
MTN	ASO	0.94625006						
AId	TT	0.94615173						
SO	PH	0.945965						
AUDp	VISal	0.94594586						
AUDd	AUDp	0.94558539						
MPN	LHA	0.94551754						
CA3	SFO	0.94551285						
BLA	RCH	0.94548918						
GPi	PVH	0.94541751						
PVa	PSTN	0.94533537						
VENT	RCH	0.94491126						
FS	MPO	0.94457886						
VISl	RAmb	0.94456715						
CLA	PS	0.9443564						
LA	ZI	0.94422114						
BA	GPi	0.94383565						
BA	SPF	0.94379759						
DMH	AHN	0.94359867						
VENT	ATN	0.9431805						
MPN	LPO	0.94311174						
IG	EP	0.94298431						
VENT	LPO	0.9427567						
PVa	SBPV	0.9424615						
AUDpo	PL	0.9422474						
VISl	PL	0.94210523						
BLA	ZI	0.94190109						
MTN	PVH	0.94189211						
AAA	PVH	0.94180615						
SPF	SFO	0.94180593						
BST	ZI	0.94164204						
AAA	LHA	0.94161134						
SCH	VMH	0.941403						
DTN	LDT	0.9413247						
PVi	AHN	0.94123473						
B	PCG	0.94083108						
MTN	ZI	0.9407045						
PBG	y	0.94057951						
GPi	SO	0.94046233						
VISpl	RPO	0.94032944						
NLOT	VENT	0.94024287						
GPi	VMH	0.94015375						
ILM	LHA	0.94014749						
IA	SO	0.94009384						
ATN	ZI	0.94005974						
BLA	TRS	0.93967564						
VLPO	LPO	0.93953464						
GPi	BST	0.93943156						
PBG	VTN	0.939317						
CEA	ZI	0.93914887						
PSV	RM	0.93908893						
GPi	ATN	0.93896587						
FS	PVH	0.93864564						
CEA	IA	0.93859169						
GENv	SO	0.93854332						
FS	BA	0.93843587						
NLOT	GPi	0.93837297						
BA	SPA	0.93834738						
CA3	RT	0.93785128						
BA	STN	0.93765381						
IA	LPO	0.93741348						
CA3	ILM	0.93735763						
LA	TU	0.93725814						
NLOT	BMA	0.93709655						
GPi	MTN	0.9369888						
LAT	MTN	0.93690998						
SFO	AHN	0.93685218						
SPA	MPN	0.93640551						
SSp	ATN	0.93628147						
LHA	LPO	0.93619244						
AUDp	AUDv	0.9361108						
SPF	LPO	0.93598856						
NLOT	AAA	0.93593061						
LA	GENv	0.93568661						
IG	SF	0.93561799						
FC	PAS	0.93541275						
RT	SO	0.93540938						
BA	ATN	0.93520006						
ASO	PVH	0.93518927						
LPO	ZI	0.93501205						
PVH	ZI	0.93486845						
GENv	LPO	0.93485454						
CA3	ATN	0.93483265						
SPA	VLPO	0.93477788						
CA2	LA	0.93438468						
AUDv	ENT	0.93417613						
AIp	NLOT	0.93402644						
LA	VENT	0.93369715						
CA3	GENv	0.93358751						
SPF	LHA	0.93354676						
CEA	PVH	0.93347423						
ASO	LPO	0.93328741						
ATN	PVH	0.93306051						
VENT	SPA	0.93272476						
ARH	SCH	0.93271344						
ACVII	LRN	0.93231827						
CA3	IA	0.93230107						
LA	BLA	0.9321628						
CEA	LPO	0.93214947						
LA	SBPV	0.93213559						
GPi	LPO	0.93189661						
BA	ZI	0.93183444						
BLA	GPi	0.93174762						
SO	AHN	0.93168775						
CEA	PS	0.93145403						
FS	ILM	0.93140978						
PVa	VMH	0.93140772						
MPO	VLPO	0.93136452						
COA	PAA	0.93131347						
LA	AHN	0.93126611						
AHN	LPO	0.9310919						
LAT	MED	0.9310646						
TRS	LHA	0.9310111						
TRS	TU	0.93099284						
SO	ZI	0.93098661						
HEM	PVi	0.93087907						
SO	LHA	0.93051935						
BLA	SF	0.93041925						
MA	MPN	0.93027973						
SPF	ILM	0.93024165						
BST	VENT	0.93017928						
BST	SPF	0.93014724						
MA	SO	0.93013266						
CA3	SPF	0.9300586						
PVH	PH	0.92999176						
MTN	LPO	0.92993105						
CEA	TU	0.92979948						
ORBl	IG	−0.9309675						
IG	ENT	−0.9313086						
TEa	IG	−0.932185						
TT	TU	−0.9326137						
B	IO	−0.9330159						
AId	LA	−0.9338227						
VISl	ACB	−0.933958						
TT	RCH	−0.9356735						
PRE	PA	−0.9372874						
PRE	VMH	−0.9379279						
ORBl	SF	−0.9411831						
TT	BMA	−0.9411929						
AUDpo	LS	−0.9433956						
PRE	RCH	−0.9444777						
ORBm	SF	−0.9455869						
AOB	SPF	−0.9474364						
PRE	BST	−0.949783						
TEa	EP	−0.9497843						
ORBm	CP	−0.9506136						
PL	LS	−0.9512804						
ENT	EP	−0.9522718						
DP	BLA	−0.9606578						
AUDp	LS	−0.9614194						
ORBl	CA2	−0.9640302						
TT	CA2	−0.9653638						
TT	BLA	−0.9756476						

**Table 6:** Sensitivity analysis. Results from permutation testing (n = 1,000 permutations) pairwise group-level differences in network density, using different thresholding methods to derive pup call response networks from ***cFos^PC^*** correlation matrices.

Naïve virgins vs. Experienced virgins			Naïve virgins vs. Mothers			Experienced virgins vs. Mothers		
*Edge* *thresh*.	*Obs. Δ* *network* *density*	*p-value*	*Edge* *thresh*.	*Obs. Δ* *network* *density*	*p-value*	*Edge* *thresh*.	*Obs. Δ* *network* *density*	*p-value*
p < 0.05	0.028429	0.106 (ns)	p < 0.05	0.048685	0.019 (*)	p < 0.05	0.020256	0.128 (ns)
p < 0.01	0.018735	0.069 (ns)	p < 0.01	0.028689	0.021 (*)	p < 0.01	0.0099544	0.17 (ns)
p < 0.001	0.0069115	0.136 (ns)	p < 0.001	0.010433	0.059 (ns)	p < 0.001	0.003521	0.226 (ns)
FDR-BH (α = 0.05)	0.013128	0.095 (ns)	FDR-BH (α = 0.05)	0.016257	0.037 (*)	FDR-BH (α = 0.05)	0.0031298	0.307 (ns)
FDR-BKY (α = 0.05)	0.012997	0.094 (ns)	FDR-BKY (α = 0.05)	0.01591	0.038 (*)	FDR-BKY (α = 0.05)	0.0029124	0.309 (ns)

**Table 7:** GLMM results. Results from fitting a full and reduced GLMM (see Methods) per brain region, and comparing the fit of the 2 models. Significant p-value would indicate that full GLMM explains the OXT+ voxel counts in that brain region better than the reduced GLMM. Middle column indicates p-value of model comparison (green shading highlights regions with p < 0.05), rightmost column indicates adjusted p-value of model comparison (red shading highlights regions with FDR < 0.10).

Region	p-value from model comparison	Adjusted p-value (FDR < 0.10)
FRP	0.88463197	0.97038711
MOp	0.53115262	0.87010215
MOs	0.81183505	0.96744583
SSp	0.2650033	0.76072212
SSs	0.04433771	0.59578798
ILA	0.4453256	0.85534112
GU	0.55649087	0.87010215
VISC	0.85969596	0.96744583
AUDd	0.69844534	0.92845449
AUDp	0.12339947	0.66128526
AUDpo	0.03574206	0.59578798
AUDv	0.12616597	0.66128526
VISal	0.00352761	0.37921769
VISam	0.70160869	0.92845449
VISl	0.11146917	0.66128526
VISp	0.16660817	0.68886069
VISpl	0.0533417	0.64336192
VISpm	0.77079325	0.95125265
ACAd	0.45672744	0.86444671
ACAv	0.98997561	0.99927115
PL	0.49329965	0.87010215
ORBl	0.31788528	0.82035051
ORBm	0.0913891	0.66128526
ORBvl	0.15352374	0.66255375
AId	0.62882562	0.88945729
AIp	0.53248062	0.87010215
AIv	0.49464937	0.87010215
RSPagl	0.47604108	0.87010215
RSPd	0.32665885	0.82035051
RSPv	0.21298983	0.74607731
TEa	0.10662704	0.66128526
PERI	0.49496162	0.87010215
ECT	0.22325802	0.74607731
MOB	0.24898863	0.74607731
AOB	0.00340371	0.37921769
AON	0.18805144	0.7219832
TT	0.24984915	0.74607731
DP	0.85519355	0.96744583
PIR	0.12653861	0.66128526
NLOT	0.36944157	0.82035051
COA	0.35984535	0.82035051
PAA	0.09023485	0.66128526
TR	0.00861251	0.43152577
CA1	0.50104694	0.87010215
CA2	0.87745087	0.96744583
CA3	0.72545167	0.93475934
DG	0.73246751	0.93475934
FC	0.0672615	0.66128526
IG	0.09625729	0.66128526
ENT	0.02988062	0.59578798
PAR	0.34888389	0.82035051
POST	0.76776163	0.95125265
PRE	0.03322533	0.59578798
SUB	0.31521928	0.82035051
CLA	0.33971307	0.82035051
EP	0.49026998	0.87010215
LA	0.68194001	0.92795635
BLA	0.60300102	0.87010215
BMA	0.90291996	0.97718426
PA	0.46685123	0.87010215
CP	0.12252117	0.66128526
ACB	0.50943987	0.87010215
FS	0.75435714	0.94846073
OT	0.65055124	0.9006051
LS	0.41367077	0.83007681
SF	0.04185403	0.59578798
SH	0.10662301	0.66128526
AAA	0.69770583	0.92845449
BA	0.30438002	0.82035051
CEA	0.24762345	0.74607731
IA	0.71528073	0.93203247
MEA	0.06861947	0.66128526
GPe	0.40512507	0.82415358
GPi	0.40632688	0.82415358
SI	0.03476809	0.59578798
MA	0.14784291	0.66255375
MSC	0.37011162	0.82035051
TRS	0.01003548	0.43152577
BST	0.06883298	0.66128526
BAC	0.5611744	0.87010215
VERM	0.13278187	0.66128526
HEM	0.55995953	0.87010215
FN	0.53868818	0.87010215
IP	0.79261735	0.95971508
DN	0.74709564	0.94485625
VENT	0.15008326	0.66255375
SPF	0.09388679	0.66128526
SPA	0.90900861	0.97718426
PP	0.54576201	0.87010215
GENd	0.96253946	0.99493261
LAT	0.99974091	0.99974091
ATN	0.98296291	0.99927115
MED	0.66490262	0.91053543
MTN	0.43705281	0.85423959
ILM	0.94916273	0.99493261
RT	0.73216501	0.93475934
GENv	0.99480946	0.9994581
EPI	0.22405525	0.74607731
SO	0.84460907	0.96744583
ASO	0.15939555	0.67196165
PVH	0.12608408	0.66128526
PVa	0.09779936	0.66128526
PVi	0.08060429	0.66128526
ARH	0.61594702	0.8828574
ADP	0.23489165	0.74607731
AVP	0.95610281	0.99493261
AVPV	0.64613085	0.9006051
DMH	0.5043899	0.87010215
MEPO	0.56269805	0.87010215
MPO	0.40397505	0.82415358
OV	0.27961256	0.78529472
PD	0.58146625	0.87010215
PS	0.23557755	0.74607731
PVp	0.77254559	0.95125265
PVpo	0.11979533	0.66128526
SBPV	0.35282531	0.82035051
SCH	0.3598652	0.82035051
SFO	0.31286682	0.82035051
VLPO	0.98787803	0.99927115
AHN	0.39403858	0.82415358
MBO	0.54711792	0.87010215
MPN	0.29779322	0.82035051
PMd	0.24044945	0.74607731
PMv	0.62448619	0.88916908
PVHd	0.52609149	0.87010215
VMH	0.33280264	0.82035051
PH	0.59693365	0.87010215
LHA	0.82934272	0.96744583
LPO	0.09963081	0.66128526
PST	0.58075761	0.87010215
PSTN	0.39745404	0.82415358
RCH	0.90869235	0.97718426
STN	0.96077852	0.99493261
TU	0.9541251	0.99493261
ZI	0.40015679	0.82415358
SCs	0.48460134	0.87010215
IC	0.31542643	0.82035051
NB	0.25535839	0.75208292
SAG	0.00647539	0.43152577
PBG	0.94770284	0.99493261
MEV	0.05572317	0.64336192
SNr	0.45835779	0.86444671
VTA	0.58798278	0.87010215
RR	0.35413132	0.82035051
MRN	0.79455481	0.95971508
SCm	0.41696882	0.83007681
PAG	0.85212583	0.96744583
PRT	0.35594239	0.82035051
CUN	0.97007192	0.99792088
RN	0.24852848	0.74607731
III	0.14544031	0.66255375
EW	0.36791604	0.82035051
IV	0.22736713	0.74607731
VTN	0.82409942	0.96744583
AT	0.65346231	0.9006051
LT	0.87319686	0.96744583
SNc	0.24531827	0.74607731
PPN	0.53083842	0.87010215
RAmb	0.38713104	0.82415358
NLL	0.34019588	0.82035051
PSV	0.93564126	0.99493261
PB	0.22060215	0.74607731
SOC	0.86780225	0.96744583
B	0.01722509	0.52905632
DTN	0.13217395	0.66128526
PCG	0.03930586	0.59578798
PG	0.12758925	0.66128526
PRNc	0.13184129	0.66128526
SG	0.04068224	0.59578798
SUT	0.5950596	0.87010215
TRN	0.1353328	0.66128526
V	0.58817856	0.87010215
CS	0.86286482	0.96744583
LC	0.18213445	0.71198013
LDT	0.03886348	0.59578798
NI	0.42729406	0.84282774
PRNr	0.21500252	0.74607731
RPO	0.60003824	0.87010215
SLC	0.51081719	0.87010215
SLD	0.15408227	0.66255375
AP	0.52741678	0.87010215
CN	0.65278955	0.9006051
DCN	0.3998506	0.82415358
ECU	0.57344843	0.87010215
NTB	0.32171754	0.82035051
NTS	0.77869984	0.95125265
SPVC	0.77833661	0.95125265
SPVI	0.0143999	0.51599624
SPVO	0.05685524	0.64336192
VI	0.28124508	0.78529472
VII	0.17054689	0.69184115
ACVII	0.83889517	0.96744583
AMB	0.19617655	0.73996418
DMX	0.15155161	0.66255375
GRN	0.37873254	0.82415358
ICB	0.86971068	0.96744583
IO	0.89653114	0.97718426
IRN	0.71174169	0.93203247
ISN	0.69543189	0.92845449
LIN	0.26536818	0.76072212
LRN	0.98757939	0.99927115
MARN	0.81311542	0.96744583
MDRN	0.86548333	0.96744583
PARN	0.70389806	0.92845449
PAS	0.09206406	0.66128526
PGRN	0.53391791	0.87010215
PHY	0.86486487	0.96744583
PPY	0.568148	0.87010215
VNC	0.20864546	0.74607731
x	0.91988361	0.9839551
XII	0.44557305	0.85534112
y	0.54755158	0.87010215
RM	0.17989375	0.71198013
RPA	0.82168412	0.96744583
RO	0.73476432	0.93475934

**Table 8:** Relative OXT projection density per brain region, per group. For each of the 215 brain regions conserved from Allen Brain Atlas, this table lists their abbreviation (see Table 1 for abbreviations) and relative OXT projection density (see Methods) per group, in descending degree order.

Region abbrev.	Relative OXT density
SO	0.2398735
PVpo	0.2272197
OV	0.2224475
PS	0.2040859
PD	0.1780798
PVa	0.1451227
PVH	0.0954982
AVPV	0.0902969
MEPO	0.0807859
VLPO	0.0625103
MPO	0.0512122
LC	0.044878
PPY	0.0444063
AVP	0.0432376
BST	0.0371417
SBPV	0.0354927
SCH	0.0335197
MEV	0.0312559
TU	0.0311497
PVi	0.0295441
ORBm	0.0256713
ARH	0.0254955
ADP	0.0248438
MPN	0.02389
RPA	0.0238859
PVHd	0.0183432
DMH	0.0179946
RCH	0.0176264
ASO	0.0164545
ORBl	0.0162405
B	0.0151445
PB	0.0149309
DMX	0.0141134
PBG	0.0140608
PRE	0.0137669
RR	0.0136902
XII	0.0127117
BAC	0.0126811
PVp	0.012131
LPO	0.0111976
PCG	0.0110871
RM	0.0110693
ORBvl	0.0099243
IV	0.008913
PMd	0.0087054
LS	0.0083908
AIv	0.0083629
MSC	0.008261
LHA	0.0081678
DTN	0.0080212
SAG	0.0079773
SPA	0.0078994
RAmb	0.0074641
MARN	0.0072059
LIN	0.0070781
VTA	0.0068938
RO	0.006873
SLD	0.0065803
TT	0.0059825
POST	0.0057253
LRN	0.0057019
AHN	0.0056907
NTS	0.0056267
CEA	0.0055895
LDT	0.0053934
PGRN	0.0048776
AAA	0.0045777
MEA	0.0045748
VNC	0.0045329
AIp	0.0044675
PH	0.0044538
SNc	0.0044104
PMv	0.0043906
FRP	0.0043282
PPN	0.0040999
PSTN	0.0040628
ICB	0.0040547
SI	0.0040161
PAR	0.0039526
ACAd	0.0039449
PAG	0.0038432
ISN	0.0038281
NTB	0.0037293
MBO	0.0037139
y	0.003645
VERM	0.0035691
SLC	0.0035162
PL	0.0035111
VMH	0.0034696
MRN	0.003465
PHY	0.0034305
MTN	0.0034128
PAS	0.0034093
AON	0.0032142
AP	0.0032085
PST	0.0031069
SCs	0.003067
SPF	0.0029877
SF	0.0028322
ACB	0.0027295
CUN	0.0027034
IC	0.0026972
NB	0.0026414
PG	0.0025486
PP	0.0024559
OT	0.0022886
DP	0.0022078
VISC	0.0021653
PRNr	0.0021224
IO	0.002048
BA	0.0020092
LT	0.002001
SFO	0.0019641
MA	0.0019138
TRN	0.001859
GENd	0.0018462
CN	0.0017355
MOB	0.0017313
ECU	0.0017302
PERI	0.0016235
SSs	0.0015439
SNr	0.0015325
STN	0.0015102
NLL	0.0014843
AId	0.0014463
PRNc	0.0014373
RSPv	0.0013792
SUT	0.0013643
VISam	0.0013569
RSPd	0.0013038
RSPagl	0.0012922
x	0.001287
MOs	0.001271
ECT	0.0012055
EPI	0.0012025
ILA	0.0011828
FC	0.0011344
DG	0.0011016
PIR	0.0010732
IA	0.0010622
AT	0.0010605
CLA	0.0010523
SSp	0.0010491
AOB	0.0010487
FN	0.0010486
NLOT	0.0010377
GRN	0.0010322
CA2	0.0010266
FS	0.0009729
CP	0.0008866
RN	0.0008524
VISpm	0.0008494
TRS	0.0008033
ATN	0.0007927
MDRN	0.0007889
GPi	0.000762
VTN	0.0007555
MOp	0.0007461
ZI	0.000738
NI	0.0007105
V	0.0006944
DN	0.0006426
MED	0.0006304
SH	0.0006256
TEa	0.0006225
HEM	0.0006104
VISp	0.0006
EP	0.0005956
LA	0.000587
PARN	0.0005744
IRN	0.0005649
IP	0.0005376
GU	0.0005371
ILM	0.0004652
VI	0.0004495
SPVC	0.0004476
DCN	0.0004311
RPO	0.0004195
SUB	0.0004106
PAA	0.0004
SPVO	0.0003997
EW	0.0003919
RT	0.0003894
AUDpo	0.0003879
COA	0.0003864
CA3	0.0003838
AUDv	0.0003602
AUDp	0.0003529
BMA	0.0003346
PRT	0.0003319
VII	0.0003284
GPe	0.0003213
GENv	0.000317
SCm	0.0003089
CS	0.0003044
PA	0.000303
ACVII	0.0002837
PSV	0.0002801
ENT	0.0002779
III	0.0002747
SOC	0.0002682
ACAv	0.000245
BLA	0.0002017
SPVI	0.000198
VISpl	0.0001804
AMB	0.0001583
CA1	0.000158
LAT	0.0001579
VENT	0.0001368
SG	0.000123
TR	0.0001061
VISal	8.301E-05
AUDd	8.248E-05
VISl	7.454E-05
IG	0.0000309
215	0
